# Comparison of mapping algorithms used in high-throughput sequencing: application to Ion Torrent data

**DOI:** 10.1186/1471-2164-15-264

**Published:** 2014-04-05

**Authors:** Ségolène Caboche, Christophe Audebert, Yves Lemoine, David Hot

**Affiliations:** 1 FRE 3642 Molecular and Cellular Medecine, CNRS, Institut Pasteur de Lille and Univ Lille Nord de France, Lille, France; 2 Genes Diffusion, 3595, Route de Tournai, 59501 Douai, France; 3 Transcriptomics and Applied Genomics, Center of Infection and Immunity of Lille, Inserm U1019, CNRS UMR8204, Institut Pasteur de Lille, Univ Lille Nord de France, Lille, France; 4 PEGASE-Biosciences, Institut Pasteur de Lille, 1 Rue du Professeur Calmette, 59019 Lille, France

**Keywords:** High-throughput sequencing, Mapping algorithms, Mapper comparison, Read simulator

## Abstract

**Background:**

The rapid evolution in high-throughput sequencing (HTS) technologies has opened up new perspectives in several research fields and led to the production of large volumes of sequence data. A fundamental step in HTS data analysis is the mapping of reads onto reference sequences. Choosing a suitable mapper for a given technology and a given application is a subtle task because of the difficulty of evaluating mapping algorithms.

**Results:**

In this paper, we present a benchmark procedure to compare mapping algorithms used in HTS using both real and simulated datasets and considering four evaluation criteria: computational resource and time requirements, robustness of mapping, ability to report positions for reads in repetitive regions, and ability to retrieve true genetic variation positions. To measure robustness, we introduced a new definition for a correctly mapped read taking into account not only the expected start position of the read but also the end position and the number of indels and substitutions. We developed CuReSim, a new read simulator, that is able to generate customized benchmark data for any kind of HTS technology by adjusting parameters to the error types. CuReSim and CuReSimEval, a tool to evaluate the mapping quality of the CuReSim simulated reads, are freely available. We applied our benchmark procedure to evaluate 14 mappers in the context of whole genome sequencing of small genomes with Ion Torrent data for which such a comparison has not yet been established.

**Conclusions:**

A benchmark procedure to compare HTS data mappers is introduced with a new definition for the mapping correctness as well as tools to generate simulated reads and evaluate mapping quality. The application of this procedure to Ion Torrent data from the whole genome sequencing of small genomes has allowed us to validate our benchmark procedure and demonstrate that it is helpful for selecting a mapper based on the intended application, questions to be addressed, and the technology used. This benchmark procedure can be used to evaluate existing or in-development mappers as well as to optimize parameters of a chosen mapper for any application and any sequencing platform.

## Background

High-throughput sequencing (HTS) technology has recently shown a rapid and impressive development and this has led to the production of gigabases of sequence in a few hours for only a fraction of the former cost [1]. HTS has produced an explosion of knowledge in genetics and genomics thanks to the development of specific applications such as genome re-sequencing (whole genome sequencing and targeted sequencing). This technological evolution was paralleled by the development of new algorithms to deal with the quantity and the quality of reads produced. A fundamental analysis steps in re-sequencing approaches is the mapping of the reads onto a reference genome. This step, which involves the accurate positioning of reads onto a reference genome sequence, is highly important because it determines the global quality of downstream analyses. The algorithms used for this step are called mappers. Mappers have to be sensitive and accurate and, if possible, fast and not too computationally demanding. They should be able to find the true position of each read on a reference genome and ideally distinguish between technical sequencing errors and natural genetic variations.

In recent years many mappers have been developed and distributed (more than 60 mappers are listed in [2]). Two studies [2,3] have classified mappers using a wide variety of features that include: the type of data, their application, the sequencing platform, the read length, the allowed error rate, parallel implementation, the ability to deal with multi-mapped reads (*i.e.* reads aligned to multiple locations), the input and output formats, and the available parameters. Mappers have multiplied and so has the range of possible settings. Hence, the growing difficulty in selecting a mapper has been raised in recent studies aimed at evaluating mapper performances through a multiplicity of comparison criteria. Some of these studies have focused on mapper sensitivity (ability to correctly map reads) [4-6]. Schbath *et al.* studied the ability of mappers to identify unique versus multi-mapped reads using a well-controlled benchmark containing reads with exactly three mismatches [7]. Hatem *et al.* introduced a benchmarking suite to analyze mapping tools [8], which consists of tests that cover input properties and algorithmic features.

In addition to the difficulty in determining evaluation criteria, choosing an appropriate evaluation method, *i.e.* how to compare mappers according to the evaluation criteria, and using the appropriate metrics, are also problematical. Using real datasets to evaluate mapper performances allows only a rough assessment and classification of mappers by comparing the percentage of mapped reads, but does not reveal the actual accuracy of mappers. Attempts have been made to avoid this pitfall using simulated datasets in which the original read positions are known. Another difficulty lies in the accurate definition of what a correctly mapped read is. The basic definition is to consider a read as correctly mapped if the original location is retrieved [4]. Ruffalo *et al.* broadened this definition by adding a condition on the quality score, which had to be superior to a given threshold [5]. In a more recent paper [8], a new definition was introduced in which a read was considered to be correctly mapped if the mapping criteria were not violated, *i.e.* contained less errors than the threshold parameter set by the user.

Using simulated data allows numerical values to be obtained and compared between a set of mappers. However, simulated data do not have the same characteristics as real data, even when an error model based on real data is used. Real HTS data present biases [9] that can be very difficult to simulate. Additionally, the current definition of the mapping correctness based only on the original start location presents some weaknesses: a read can have several correct positions on the reference sequence and sequencing errors or true genetic variations can lead to a better alignment in a genome position different from the original one. Holtgrewe *et al.* introduced the interval definition, rather than the genome position, to describe a read mapping [6] and used a full-sensitivity algorithm to identify all possible matching intervals within a given error rate range for each read. This method has been implemented in RABEMA (Read Alignment BEnchMArk), a tool that evaluates the result of arbitrary read mappers that support the SAM output format with real and simulated datasets. Our analysis of the published literature on mapper evaluation led us to conclude that for a complete and robust comparison of mappers, real and simulated datasets should be used. Using real datasets avoids simulation biases and gives a real picture of mapper behavior, whereas simulated datasets are benchmarks from which all parameters can be controlled. Additionally, a sound, more complete definition of what constitutes a correctly mapped read needs to be considered (see below).

In all the previous studies, mapper performance was evaluated using large eukaryotic genomes (mainly the human genome) and, for the most part, short Illumina or Illumina-like reads data were used, except in [4,6] where 454 datasets were evaluated with a reduced number of mappers and metrics. The type of sequencing errors and their rate is inherent to the sequencing technology and more precisely to the nucleotide elongation detection methods used. For example, Life Technologies sequencing by oligonucleotide ligation and detection (SOLiD) technology showed a strong bias in its coverage of repetitive elements [10], whereas the Illumina reversible dye-terminator sequencing technology (HiSeq) mainly caused substitutions [11]. Pyrosequencing on solid support (454/Roche) and ion semiconductor sequencing technology (Ion Torrent, Life Technologies) produced indel errors associated with homopolymer-regions [12]. In the published evaluations, the criteria that were tested and the default parameters of the mappers were usually chosen to address or deal with substitution-type errors and are, therefore, less informative for mapping the reads from new technologies like the Ion Torrent platform.

Furthermore, the analysis of small microbial genomes compared with the analysis of large eukaryotic genomes poses other challenges because microbial genomes contain a wide range of GC content, which is sometimes extreme. Very high or very low GC content means that there is a high probability of encountering homopolymers in a genome sequence and this is known to be a specific problem for pyrosequencing and ion semiconductor sequencers. A recent development in the HTS technologies has made available benchtop sequencers targeted at the quick and inexpensive sequencing of small to moderate-sized genomes, mainly bacteria, viruses, fungi, and parasites. Small microbial genome sequences could be considered to present a simpler, less demanding mapping process compared with the mapping process for larger eukaryotic genomes. However, this is only partially true because the characteristics of small microbial genomes are not the same as those of eukaryotic genomes. The questions of interest are also usually different and, consequently, the expected mapping quality criteria are not exactly the same. Whole genome sequencing or re-sequencing is an important application in the new field of microorganism characterization using HTS. For instance, clinical diagnosis and the epidemiological study of microbial strain circulation will be profoundly remodeled in the near future by the use of HTS, which should, very soon, be used as a characterization approach for pathogens and which will probably slowly replace the present PCR and biochemical based characterization methods [13,14]. In this particular context the re-sequencing applications and derived analyses are in the front-line of research and development. The focus includes the sequencing of the entire length of a microbial genome and the analysis of obtained reads by mapping them onto one or several reference strains to identify potential relevant changes in the studied genome. The aim is to accurately identify the gain or loss in genetic elements (genes or parts of genes, prophages, and plasmids) as well as small changes (mutations and indels) to predict a potential new phenotype or a derived new pathogenicity profile. This requirement poses several challenges, the most important of which is the necessity to distinguish true genetic variations from sequencing errors.

In this paper, we focus on the evaluation of mappers in the context of whole genome sequencing or re-sequencing for small microbial, mainly bacterial genomes. We tested 14 mappers, mostly using their default settings to be in the general context of non-expert users. We selected four criteria to match this context: (i) computational resource and time requirements, (ii) robustness of mapping through the evaluation of precision, recall and F-measure, (iii) ability to report positions for reads in repetitive regions, and (iv) ability to retrieve true genetic variation positions. To evaluate a mapper’s robustness on simulated datasets, we introduced a new definition of a correctly mapped read. In addition to the original start position (*i.e.* the position from which a read is simulated) that was used in most previous studies, the end position as well as the numbers of insertions, deletions, and substitutions in the alignment were also used to classify the mapping of a read as correct. This definition is more stringent than the previous ones because it implies that it is a full-length read alignment and that the error count is correct. Indeed, sequencing errors can mean that the original location of a read is not necessarily the best alignment location. Using mappers tuned to report all possible hits (‘all’ mode) and to accept a higher error rate than the error rate introduced in simulated reads, it should be possible to retrieve the original location in addition to potential equivalent or better hits. With the new definition of a correctly mapped read used in this study, we ensured that the mapper was able to retrieve the expected original alignment despite inevitable sequencing errors in the reads, thereby allowing a true evaluation of the mapper’s robustness.

The analysis was applied to data generated by the Ion Torrent Personal Genome Machine (PGM), a newly arrived technology dedicated mainly to small genome sequencing, for which mapper performances have not yet been evaluated. Reads from real datasets and artificially simulated reads were used. Simulated reads were generated using a new customizable read simulator, CuReSim, which can generate reads of user-determined lengths with insertions, deletions, and substitutions introduced at a controlled rate and with an adjustable error distribution along the read. CuReSim and CuReSimEval, a script that can be used to evaluate mapping quality, were developed in Java to run on all operating systems (see Section 2 of Additional file 1 for more details) and are freely available at http://www.pegase-biosciences.com/tools/curesim/. We have shown that in microbial genome sequencing, some mappers, such as segemehl, present higher robustness than others, especially when the number of sequencing errors was high. Other mappers are more robust for other applications that demand other quality criteria. For example, BWASW, SHRiMP2, SMALT, SSAHA2 and TMAP, might perform particularly well for sequencing focused on rare variant discovery because they show a robust discrimination of variations. SMALT can localize most of the positions of reads located in repeated regions. Some mappers, such as Novoalign, SMALT and SRmapper, needed very small memory resources (about 20 MB), while SNAP was very fast and required only about two minutes to process the bigger datasets used in this study. These results emphasize the observation that mapper choice is application dependent and users should carefully consider the targeted aim before choosing a mapper. The evaluation approach presented here, together with the developed tools (CuReSim to generate simulated reads and CuReSimEval to evaluate mapping quality) can be considered as a general method to evaluate existing or in-development mappers and could prove interesting in the evaluation of the performances of mappers for the coming third generation of sequencers that may have yet another type and rate of errors.

## Results

### Computational resource requirement and time measurement

**Figure 1:**
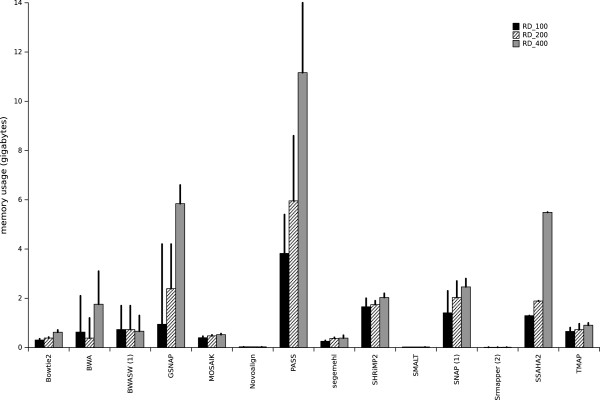
**Computational resource requirements for each mapper dealing with real datasets.** Random Access Memory (RAM, in gigabytes) consumption observed for each mapper, for the three real datasets (RD_100, RD_200 and RD_400) is shown. The values are the requirements for when the indexing and mapping steps were considered together. Vertical bars show the mean memory usage and vertical lines represent the peak memory usage. (1) indicates the mappers that report only one read (‘any-best’ mode) and (2) indicates the mappers that can run only in ‘all-best’ mode.

All mapping processes involve the alignment of millions of reads onto a reference sequence. This is true even for small genome sequencing projects where the small size of the reference sequence is generally compensated by the multiplicity of samples to be analyzed. In clinical microbiology, the time and the computational resources required for the analysis are critical; therefore, 0 these factors also need to be evaluated for the different mappers. All the mappers tested were run with 24 threads (except for Novoalign, SRmapper, and SSAHA2, which can be run with only 1 thread) and the memory consumption and runtime were recorded for three different Ion Torrent datasets RD_100, RD_200, and RD_400. These three datasets contain real single-reads with different mean sizes and are described in Table 1. The reference genome used was *Escherichia coli str. K-12 substr. DH10B* [GenBank:NC_010473] for the RD_100 and RD_200 datasets and *Escherichia coli str. K-12 substr. MG16655* [GenBank:NC_000913] for the RD_400 dataset. Figure 1 shows the memory consumption for each mapper for the real datasets when the indexing and mapping steps were considered together. Novoalign, SMALT, and SRmapper needed very low memory resources (about 20 MB). It should be noted that SRmapper was developed to run on a computer with 4 GB of RAM for genomes the size of the human genome, but, in such a case, it can be run only in ‘all-best’ mode and does not allow indels in the mapping. The Novoalign version used in this study was the free academic version that has not been implemented in parallel. A second group comprising Bowtie2, MOSAIK, and segemehl, needed less than 1 GB of RAM, while a third group, BWA, BWASW, and TMAP, needed less than 2 GB of memory. BWA had peak memory usage of 2 GB for RD_100 and of more than 3 GB for the RD_400 dataset. BWA was developed to map short reads of up to 100 bases, which may explain the high peak usage for 400-base reads. SHRiMP2, SNAP, and SSAHA2 required more RAM (about 3 GB) and SSAHA2 needed about 6 GB for the RD_400 dataset. Finally, the GSNAP and PASS mappers were highly memory-consuming; for the RD_400 dataset, GSNAP needed 6 GB of RAM, with a peak usage of 7 GB while PASS needed about 12 GB of RAM with a peak usage of 14 GB. The RAM requirement increased proportionally with the dataset size for half of the mappers tested, while for Bowtie2, BWASW, MOSAIK, Novoalign, SMALT, segemehl, SHRiMP2, and TMAP memory consumption was about the same for all dataset sizes. These experiments revealed that the computational resource requirements varied considerably among the mappers, from a few megabytes to 14 GB.

**Table 1:** Main features of the Ion Torrent Personal Genome Machine datasets used in this study

**Ion Torrent PGM data**				
**Name**	**Chip**	**Number of reads**	**Mean length**	**Organism**
RD_100	316	1,713,033	111 bp	*E. coli K-12 DH10B*
RD_200	316	2,176,492	226 bp	*E. coli K-12 DH10B*
RD_400	318	6,668,556	312 bp	*E. coli K-12 MG1655*

The datasets all contain only single-reads with different mean sizes.

**Figure 2:**
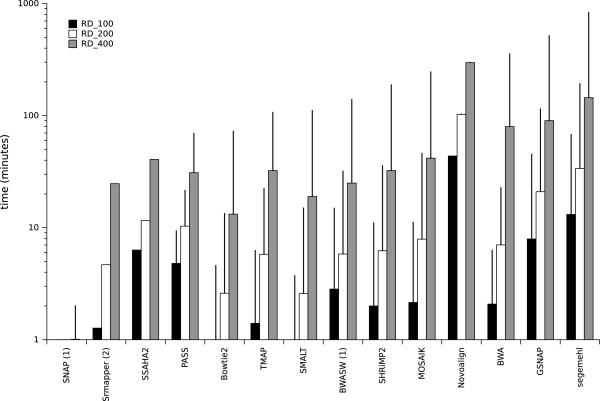
**Runtime measurements for each mapper dealing with real datasets.** Runtime measurements are in minutes. The time measurements are the runtimes for when the indexing and mapping steps are considered together. Vertical bars show the user runtime (time elapsed during the experiments) and vertical lines indicate the CPU time. The time axis is in log scale. Each mapper was run with 24 threads (except Novoalign, SRmapper, SSAHA2, which were run with 1 thread). (1) indicates the mappers that report only one read (‘any-best’ mode) and (2) indicates the mappers that can run only in ‘all-best’ mode.

The time required for the sequencing process relies mainly on the biotechnological part of the protocol (from sample preparation to the sequencing run) but the runtime of the mapping step could also constitute a bottleneck for some mappers. Figure 2 shows runtime measurements for each mapper running with the three real datasets. The mappers had very different runtimes that were not all proportional to the dataset size. SNAP was very quick and needed only about 2 minutes to map the RD_400 dataset. However, this runtime was for the program run in ‘any-best’ mode, which is always quicker than the other modes. SRmapper, SSAHA2, PASS, Bowtie2, TMAP, SMALT, BWASW, SHRiMP2, and MOSAIK needed less than 40 minutes to map the RD_400 dataset and between 1 minute (for Bowtie2 and SMALT) and 6 minutes (for SSAHA2) to map the RD_100 dataset. BWA had quick runtimes of 2 and 7 minutes for the RD_100 and RD_200 datasets, respectively, but was slower with the biggest dataset RD_400 (around 80 minutes), probably because BWA is optimized for short reads. The slowest mappers were Novoalign, GSNAP, and segemehl. The Novoalign version used in this study could only be run with one thread which explains the long runtimes observed in this study (43, 102, and 297 minutes). GSNAP runtimes were 7, 20 and 90 minutes, and segemehl needed 13, 33 and 144 minutes for the RD_100, RD_200, and RD_400 datasets, respectively. For all the mappers, the runtimes for the RD_400 dataset (which contains more reads than the other datasets) were longer. Generally speaking, the more bases in the dataset, the longer was the runtime, although the runtimes ranged from one minute to up to five hours.

### Mapper robustness

The accuracy of the sequencing technology is usually the criterion of first importance in the choice of a sequencer. Nevertheless, the mappers used to analyze the sequencing data must be able to efficiently take into account the inherent and inevitable raw data errors. A robust mapper will permit compensation for sequencing defects and will contribute to maximizing coverage while limiting noise. To evaluate mapper robustness, one method is to compute metrics (here precision, recall, and F-measure) through a benchmark formed by simulated reads for which their original location in the genome and the number and type of introduced errors are known. We used simulated datasets with varying error rates to compare mapper robustness. To avoid simulation biases, we also studied mapper robustness with RABEMA [6] using real sub-datasets.

**Figure 3:**
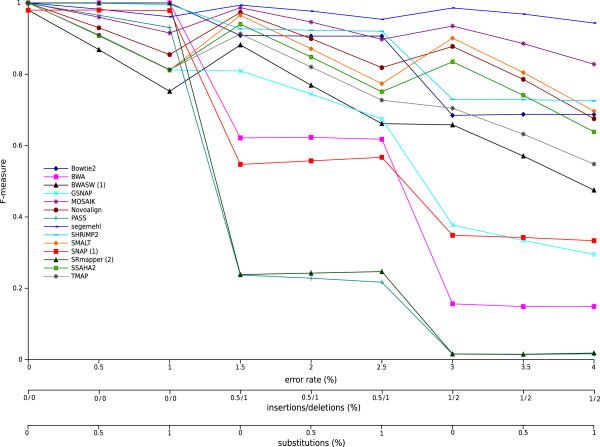
**F-measures with varying error rate for each mapper dealing with simulated datasets.** F-measures are shown for error rates from 0 to 4%. The simulated read datasets consisted of 50,000 reads with a mean length of 200 bases. (1) indicates the mappers that report only one read (‘any-best’ mode) and (2) indicates the mappers that can run only in ‘all-best’ mode.

Figure 3 shows the F-measure for each mapper with a simulated dataset containing 50,000 reads with a mean length of 200 bases and an error rate that varied from 0 to 4%. F-measure is the harmonic mean of precision and recall (see the Methods section for details). Precision is the fraction of mapped reads that are correctly mapped and recall is the fraction of correctly mapped reads that are retrieved. Additional figures that show the precision and recall values used to compute the F-measure are in Section 3.1 of Additional file 1. Figure 3 shows that the 14 mappers displayed very different robustness when the error rate increased, even when, overall, the F-measure decreased when the error rate increased. All mappers had F-measures close to 1 when the reads contained no sequencing errors or had low error rates, which meant that they were able to correctly map the whole set of reads. SRmapper, PASS, BWA, SNAP, and GSNAP showed significant decreases in the F-measure when 1.5% and 3% of indels were present in the reads. SRmapper and SNAP used with their default settings do not allow indels in the alignments, which explained the very low F-measure values observed for these two mappers. The low F-measure values for SRmapper and SNAP were attributable to low precision values, whereas for BWA, the low F-measures resulted from low recall values (see Section 3.1 in Additional file 1). Thus, with high error rates, BWA did not map a large number of reads but the mapped reads were correct; whereas, a large number of reads were incorrectly mapped by SRmapper and SNAP (see the figure showing the percentage of mapped reads in Section 3.1 in Additional file 1). The nine other mappers tested showed high F-measure values. Segemehl had a very high F-measure even with high error rates, meaning that it correctly map the major part of the read dataset. MOSAIK, SMALT, SSAHA2, and Novoalign showed peaks in the F-measure values when the dataset contained only indel errors and seemed to better handle one kind of error rather than a combination of substitution and indel errors. SHRiMP2 and Bowtie2, and more significantly TMAP and BWASW, showed a decrease of F-measure values for 1.5 and 3% indel error rates. Most of the tested mappers have been tuned mainly to deal with substitutions, which can explain their changing behaviors. The F-measure variations observed for these nine mappers are mainly the result of precision variations, except for Novoalign for which the recall values decreased at high error rates.

These experiments were repeated for simulated datasets containing reads with a mean length of 100 and 400 bases (corresponding figures can be found in Section 3.1 in Additional file 1). Overall, the F-measure values were marginally higher for the shortest reads but the mapper behaviors were similar to the behaviors observed with the dataset containing the reads with a mean length of 200 bases. However, differences were observed for BWA and GSNAP for which the F-measures were significantly better for the dataset with the shorter reads. BWA was designed to map reads up to 100 bp long, which explained the better results with short reads. The F-measures for the dataset of reads of 400 bases were lower for all the mappers and a significant decrease was observed for Novoalign, BWA (designed for short reads), and GSNAP. For the reads of 400 bases, Novoalign showed an F-measure close to 0 even when the reads contained no errors. This finding can be explained by the fact that Novoalign truncates reads before alignment (option -*n*). The maximum allowed read length is 300, so all reads longer than this are truncated to 300 before mapping.

These experiments showed that most of the mappers were less robust when the indel rate increased, probably because most mappers are tuned mainly to deal with substitutions. In the alignment step, the scoring parameters used by the mappers are often those currently used in bioinformatics, *i.e.* from the evolutionary point of view, a substitution is less penalized than an insertion or a deletion. However, in sequencing, mutations do not follow evolutionary rules; rather, they are dependent on the error model of the sequencing technology. The Ion Torrent PGM, for example, is known to introduce more indels than substitutions into homopolymer stretches. Therefore, mapper robustness could probably be improved by modifying the scoring parameters in the alignment step by decreasing the indel penalty. To test this idea, we changed the gap penalty for two mappers, SHRiMP2 and PASS. For SHRiMP2, the gap open and extension penalties were set to match the penalty for substitutions (see the Section 4.1 in Additional file 1 for more details). For PASS, a maximum gap of 8 bases was allowed with a gap open and extension penalty of 1. The F-measures that were obtained with the adapted scoring parameters behaved in the same way as previously observed for these two methods, but they were globally better for all error rates than the F-measures obtained with the default parameters (the corresponding figure can be found in Section 4.1 in Additional file 1).

All the simulated datasets described above contained 2,500 random reads (*i.e.* reads that were generated by choosing randomly a nucleotide for each position), which could not be mapped onto the reference genome. All the mappers, except SMALT and TMAP, returned all the random reads as unmapped. For SMALT and TMAP, the longer the read length the higher the number of mapped random reads. SMALT mapped only a small number of the random reads (less than 10 reads with around 30 matches), whereas TMAP mapped around 10%, 12%, and 16% of the random reads in the 100, 200, and 400 bases datasets, respectively, with around 15 matches. These percentages are not negligible and indicated that the TMAP strategy (used as the default mapper in Ion Torrent analysis suite) was to map a maximum number of reads even if the mapping was not always relevant. The reported alignments for the random reads were short and could be filtered out easily, but for non-expert users these reported hits will add to the complexity of the read mapping task.

In conclusion, most of the tested mappers were robust with low error rates. Segemehl showed the best F-measures even for datasets with high error rates and for all read lengths considered in this study. MOSAIK, SMALT, SSAHA2, Bowtie2, and SHRiMP2 correctly mapped a major part of the read datasets. The results also showed that to handle Ion Torrent reads, mappers need to allow indels in the alignments, as was clear for all tested mappers except for SRmapper and PASS with their default settings. We also demonstrated that decreasing the gap penalties could improve the mapping results for Ion Torrent data.

To avoid simulation biases, RABEMA was used to evaluate mapper performances with real datasets.

**Figure 4:**
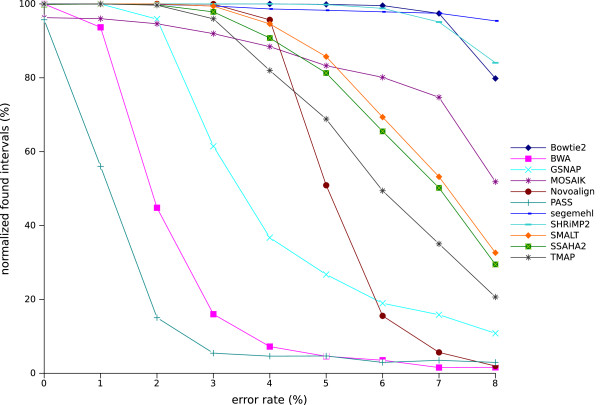
**Normalized found intervals with varying error rate for the mappers that can run in ‘all-mode’.** The percentage of normalized found intervals was obtained using RABEMA, with error rates varying from 0 to 8% for the 11 mappers run in ‘all-mode’. Mappers were run with real sub-datasets containing 50,000 reads randomly extracted from the RD_200 dataset. Each point is the mean value of four sub-datasets.

In RABEMA, a full-sensitivity algorithm was used to identify all possible matching intervals within a given error rate range for each read and the mapper evaluation was based on a metric called normalized found intervals (NFI), in which each interval for a read contributed 1/*x* points, where *x* is the number of alignments for the read. The number of points was divided by the number of reads and multiplied by 100 to get the percentage. Figure 4 shows the percentage of NFI for mappers run in the ‘all’ mode with varying error rates. Only 11 mappers were considered because BWASW, SNAP, and SRmapper cannot be run in ‘all’ mode. All the mappers identified between 100% and 95% of the NFI for datasets with no errors. However, for datasets with errors, the NFI fell rapidly to below 10% of NFI for some mappers (PASS, BWA, and GSNAP), while others (TMAP, SSAHA2, SMALT, MOSAIK, and Novoalign) maintained a high NFI percentage for datasets with up to a 4% error rate and finished at between 50 and 20% NFI for an 8% error rate (Novoalign fell rapidly and finished below 10%). Only segemehl, SHRiMP2, and Bowtie2 maintained NFI above 80%, even at an 8% error rate.

The experiments were repeated with datasets that contained reads 100 and 400 bases long (figures can be found in Section 3.1 in Additional file 1). The ranking and behavior of the 11 mappers were similar to those obtained with datasets containing read lengths of 100, except Novoalign which was significantly better with the shorter reads. For datasets with reads 400 bases long, the behavior of most of the mappers was similar to the behavior observed with 200-base long reads but the NFI percentages were lower. The Novoalign plot with several increases and decreases was atypical and only around 16% of the 400-base reads were mapped, probably because Novoalign trims reads to a maximum length of 300 bases. BWA identified around 100% NFI in the 400-base reads dataset with no errors, while with an error rate of 8% the NFI only fell to 40%. This behavior for BWA was surprising when compared with its behavior in the previous experiments; however, it can be explained by the definition of NFI used by RABEMA. In RABEMA, reads do not have to be aligned over their entire length to be considered as correctly mapped; so, many of the short alignments returned by BWA were classified as correct by RABEMA, which was not the case with our new definition. The analysis of mapper performances on real datasets with RABEMA indicated that Bowtie2, segemehl, and SHRiMP2 were better than the other mappers, even for datasets with high error rates and regardless of the read lengths.

Similar observations and similar rankings were obtained with the real and simulated datasets. This double strategy built our confidence in the conclusions drawn from these experiments and confirmed that our simulator generated reads that were similar to sequencer generated reads (at least for Ion Torrent generated reads).

### Study of repeats

**Figure 5:**
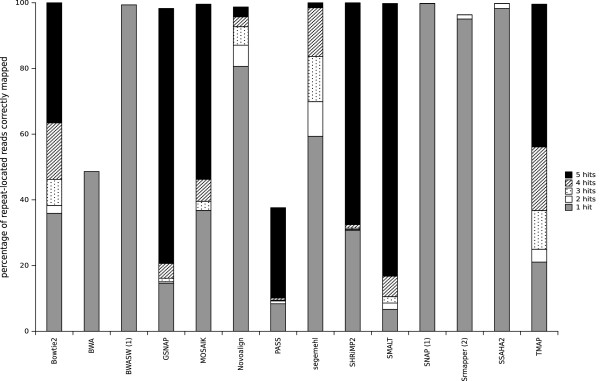
**Percentage of repeat-located reads correctly reported by the mappers.** The percentage of reads correctly reported in a repeat is shown for the mappers dealing with simulated reads of 200 bases, subdivided in classes depending on the number of identified hits. (1) indicates mappers that that report only one read (‘any-best’ mode) and (2) indicates the mappers that can run only in ‘all-best’ mode.

The study and analysis of repeated sequences is as important for small microbial genomes, especially for bacterial genomes, as it is for eukaryotic genomes. Repeats in bacterial genomes represent a smaller proportion of the total genomic DNA that they do in eukaryotic genomes, but the repeated elements are usually longer(for example, copies of homologous genes, inserted sequences, and transposons). Mapper behavior when dealing with repetitive regions in a reference genome is, therefore, an important parameter when the DNA repeat regions may also be informative regions. To study the ability of a mapper to report all possible positions for a read in a repeated sequence, we used an artificial genome containing five repeats. In theory, a mapper, in ‘all’ mode, must report 5 hits for each repeat-located read. Figure 5 shows the percentage of repeat-located reads correctly reported by the mappers with reads of 200 bases, subdivided in classes depending on the number of hits found. For each of the repeat-located reads, the number of locations in a repeat were counted. Note that BWASW and SNAP can report only one hit (‘any-best’ mode) and SRmapper is limited to all-best hits. Most of the mappers were able to map repeat-located reads in at least one repeat (percentages were close to 100%), except for BWA and PASS. Only two mappers (SMALT and GSNAP) retrieved a large proportion (more than 80%) of the 5 hits and four few others (SHRiMP2, MOSAIK, TMAP, and Bowtie2) retrieved an average proportion of the 5 hits (between 70 and 35%). The other mappers performed quite poorly in this task, retrieving only a small percentage or none of the 5 hits. With 100-base and 400-base reads, the mappers gave better and worse global results, respectively, than they did with the 200-base reads (except for TMAP which was less efficient with the 100-base reads than it was with the 200-base reads; see Section 3.2 in Additional file 1). In conclusion, SMALT was very good at retrieving multi-mapped reads whatever the read length, while GSNAP, MOSAIK, and SHRiMP2 also gave correct results. TMAP was better with longer reads and Novoalign was better with shorter reads. Mappers that cannot be run in ‘all-mode’ or that are not able to deal with indels (BWASW, SNAP, PASS, and SRmapper) are not suitable for identifying multi-mapped reads.

### Mutation discovery

**Figure 6:**
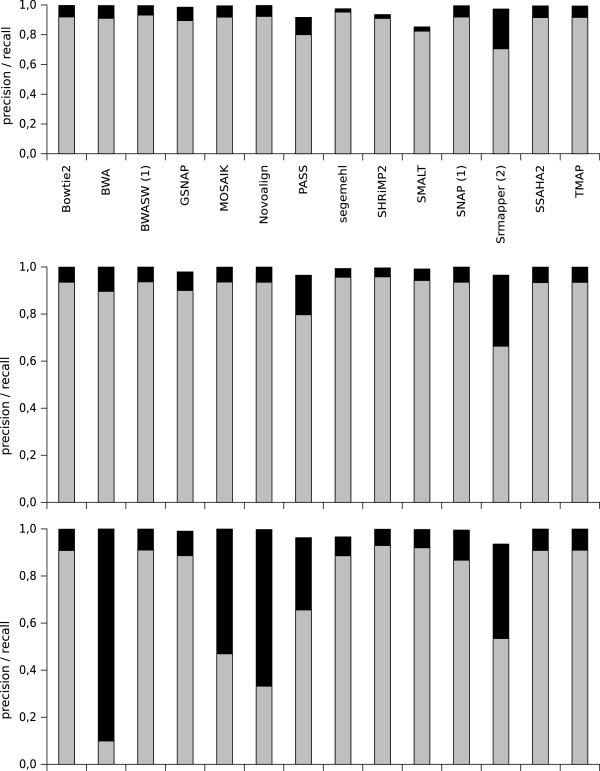
**Precision and recall values for mutation discovery with varying mutation rates in the reference genome.** The real datasets that were used contained reads of 200 bases and had a theoretical depth of 40*X*. The precision (in black) and recall (in gray) values obtained for mutation discovery for each mapper are shown. Top panel: 0.05% mutations in the reference genome; middle panel: 1% mutations in the reference genome; and bottom panel: 5% mutations in the reference genome. (1) indicates the mappers that report only one read (‘any-best’ mode) and (2) indicates the mappers that can run only in ‘all-best’ mode.

Distinguishing between sequencing or mapping errors and true genetic variations is a challenge in variant analysis. Exome sequencing and genome re-sequencing require robust mapping results with as little noise as possible to identify a mutation of interest and to limit false positive mutations. Real reads from *E. coli DH10B* sequencing were mapped onto a genome sequence in which mutations with known positions and types (substitution or indel) had been introduced artificially. FreeBayes software [15] was used to call variants, and precision and recall values were computed for mutation discovery in a reference genome with varying mutation rates. Figure 6 shows the precision and recall values obtained for mutation discovery with real datasets containing reads of 200 bases and a theoretical depth of 40*X*. Generally, precision and especially recall decreased when the mutation rate was increased in the reference genome. In all the experiments, the precision values were high, indicating that the mutations predicted by the variant caller from the mapping files were mainly correct for all mappers. Most of the tested mappers presented good precision and recall values for all mutation rates; the exceptions were BWA, Novoalign, PASS and SRmapper. SRmapper and PASS presented lower precision and recall values than all the other mappers mostly because these two mappers do not allow for indels in the alignments, which decreased the precision of the mapping (see the subsection Mapper robustness) and made the variant calling less accurate. The mutation discovery performances of BWA and Novoalign diminished when the mutation rate reached 5%. It should be noted that for these two mappers, the percentage of mapped reads, and therefore the mean depth, was low compared with the percentage of mapped reads for the other mappers (15% for BWA and 50% for Novalign - see the corresponding figure in Section 3.3 in Additional file 1). This reduced number of mapped reads did not permit the accurate detection of mutations in the reference genome. ROC curves were constructed (see the corresponding figures in Section 3.3 in Additional file 1), which confirmed the mutation discovery results that we obtained.

The experiments were repeated with simulated datasets (the corresponding figure can be found in Section 3.3 in Additional file 1). The conclusions that were drawn were similar to those obtained with the real datasets; however, the precision and recall values were lower for all mappers. We also performed similar experiments with real and simulated datasets for read lengths of 100 and 400 bases (see Section 3.3 in Additional file 1 for corresponding figures). Mapper behavior was similar regardless of the read length, except for BWA and Novoalign. These two mappers showed better values with reads of 100 bases, and showed near zero recall values with reads of 400 bases. These results were not surprising because BWA was designed for short reads and Novoalign truncates reads to a maximum length of 300 bases.

The behavior of the mappers in variant discovery was coherent with the results obtained in the robustness study and could be deduced from them. For example, SRmapper and BWA show a significant decrease in F-measure values when the error rate increased and similar behavior has been observed when the mutation rate was increased in the reference genomes. Variant discovery is impacted directly by the quality of the mapper alignments, *i.e.* position and type of edit operations (mismatches, insertions and deletions). The definition of a correctly mapped read introduced in this study is more stringent than for previous studies, because it takes into account the correctness of the alignment (length, number, and type of edit operations). These results demonstrated that the method we used to evaluate mapper robustness was efficient.

For the simulated data, similar behavior was observed for all the mappers and for all datasets but with lower precision and recall values than was observed for the real data. This decrease could be explained by a lower error rate in the real data than in the simulated data. We performed complementary analyses to observe the precision and recall values obtained with lower sequencing error rates (data not shown). When reads were generated without errors, the precision and recall values were close to 1. Precision and recall values were closer to the values obtained for the real dataset values when reads were generated with 0.5% deletions, 0.25% insertions, and 0.25% substitutions, suggesting that the real dataset used here contained less than 2% sequencing errors. These experiments again showed that the data simulated with CuReSim have characteristics that are similar to the real data produced by the Ion Torrent PGM.

Finally, because we used simulated data, the impact of sequencing depth in mutation discovery could be tested. We used SHRiMP2 because this mapper behaved well in the variant discovery experiments. The same procedure was applied with four different read datasets of 200 bases with mean depths of 20*X*, 80*X*, 160*X*, and 320*X* (results are shown in Table S1 of Section 4.2 in Additional file 1). The precision and recall values were lower with a mean sequencing depth of 20*X* and were equivalent for the other tested sequencing depths. These results showed that a mean sequencing depth of 40*X* was enough to call variations correctly. Increasing the depth of sequencing did not seem to improve the quality of variant calling.

These experiments showed that most of the tested mappers gave correct results in mutation discovery even when used with their default settings. The only exceptions were the BWA, Novoalign, PASS, and SRmapper mappers. SRmapper and PASS do not allow indels in alignments. These kinds of mappers should be avoided for variant calling analysis.

## Discussion

**Figure 7:**
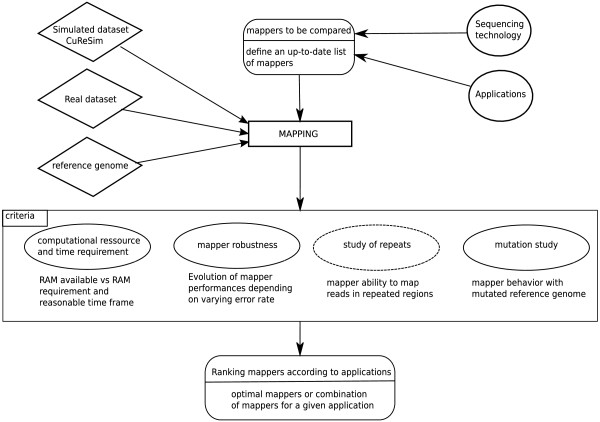
**Benchmark procedure used to compare mappers.** The different steps used to compare mappers are shown. The criteria in the solid ellipses were used with simulated and real data, whereas the criteria in the dotted ellipses were used only with simulated data.

Here, a benchmark procedure to compare mappers for HTS that can be applied to any sequencing platforms and any applications is described. The different steps involved in this procedure are shown in Figure 7. In step 1, a list of mappers is defined. Depending on the sequencing technology and the application, the most appropriate mapper can be selected for use. In step 2, real datasets are collected and simulated datasets are generated before being mapped onto the reference genome. Step 3 is a comparison step based on four criteria: mapper computational resource and time requirements; mapper robustness; mapper behavior with repetitive regions; and mapper mutation discovery ability. The benchmark procedure uses simulated and real datasets to provide the user with a robust method for mapper comparison. The results obtained can be used to answer questions such as: How much RAM is required? How long will it take to map a set of reads? How does the robustness vary in relation to the error rate? How does a mapper deal with multi-mapped reads? Could a mapper be used with a distant reference genome? What is the quality of the reported alignment? Answers to these questions can help users chose a mapper that best fits a particular application and sequencing platform. This procedure could also be used to evaluate performances of a newly developed mapper or to optimize parameters of already existing mappers.

We also presented a new read simulator, CuReSim (Customized Read Simulator), which generates synthetic HTS reads for the major letter-base sequencing platforms. Users can fix the mutation rates, the read lengths, and can generate random reads. Several error distribution modes are available and particular attention was paid to special cases in which several introduced errors in the same read can lower the number of errors because of compensatory changes. CuReSimEval is a complementary tool that evaluates the mapping quality from SAM files produced by aligning CuReSim simulated reads with any mapper. CuReSim and CuReSimEval are freely available at http://www.pegase-biosciences.com/tools/curesim/. The CuReSim suite has been developed in Java and is distributed as JAR files to be operating system independent and easy to use by non-expert users.

We used the CuReSim suite in a mapper comparison with Ion Torrent data applied to small genomes. To obtain a robust evaluation procedure, we introduced a new definition for mapping correctness. This newly introduced definition is more stringent than the previous ones because the end of the alignment and the number of mutations were considered in addition to the start position. The mapper robustness results obtained with the CuReSim suite simulated data matched the results obtained with real datasets and RABEMA, demonstrating that the CuReSim suite simulated reads with characteristics similar to real reads. We performed completely independent experiments to evaluate the mutation discovery ability of the mappers and found that the results obtained for mapper robustness can also be used to predict the mutation discovery ability of the mappers. Variant calling efficiency is directly dependent on the alignment quality obtained by the mapping algorithms. Checking whether a mapped read is in its expected position is not sufficient because the position and number of edit operations in the produced alignment must also be as close as possible to the expected alignment. The sequencing errors in Ion Torrent reads are mainly indels. For mappers that are unable to deal correctly with indels, the resulting alignments, even those at the expected positions, can result in biased mapping that could impact the variant calling results. All our results demonstrated the reliability of our evaluation method.

Our benchmark procedure was applied to Ion Torrent data from small genome sequencing. Mapping algorithms and previous mapper comparison studies focused mainly on short reads with substitutions (Illumina technology) and on large reference genomes (mainly the human genome). These evaluation studies therefore are poorly informative for mapping new technology sequencer data with different error models. Additionally, some features of bacterial and small genomes, such as possible extreme GC content, make the extrapolation from the previous studies difficult. For example, very high or low GC content percentages create a higher probability of encountering homopolymers in the genome sequence, which can significantly increase the number of indels in homopolymers. Our benchmark procedure for Ion Torrent data with bacterial genomes did not reveal a single best mapper but rather indicated several options depending on the particular application and technology. When only a desktop computer with 4 GB of RAM is available, users can select a mapper that is not highly memory-consuming; for example, Novoalign, SMALT, SRmapper, Bowtie2, MOSAIK, or segemehl. Novoalign, segemehl, and GSNAP require very long runtimes, while SNAP is very fast (around 2 minutes to deal with big datasets). Other mappers had runtimes shorter than 40 minutes for the bigger datasets. Concerning mapper robustness with varying error rates, all mappers manage to correctly map reads when the sequencing error rate was low; however, some mappers were clearly not suitable for use with datasets containing high error rates (PASS, BWA, GSNAP, SNAP, and SRmapper). Segemehl presented good F-measure values with all the tested datasets even at high error rates. MOSAIK, SHRiMP2, and Bowtie2 also gave correct results. SMALT was well fitted to retrieve all hits for repeat-located reads and GSNAP, MOSAIK, and SHRiMP2 also give correct results in this task. One of these mappers is therefore suitable for the identification of unique and non-unique reads, whereas PASS, BWA, BWASW, SNAP, and SRmapper are not. Mapper behavior for mutation discovery with datasets with varying mutation rates using a close but not identical reference genome is of special interest because often only the genome of a closely-related species is available as a reference. Mutation discovery ability is also important when the genomes of two closely-related strains are compared to detect variants or mutated strains, for example. In such cases, mappers need to produce accurate alignments so that true mutations can be detected. All the mappers tested here showed good precision and recall with all tested mutation rates and for all datasets, except BWA, Novoalign, PASS, and SRmapper.

Our results show that some mappers dealt correctly with the Ion Torrent data although they were not initially designed for this technology. For example, SHRiMP2 which was designed for Illumina, SOLiD, and 454 reads, showed robust results with Ion Torrent data.

The mapper default parameters were used deliberately in this study to mimic the general case of a non-expert user; therefore, different results could have been obtained with other parameter settings. Even with the default settings, several mappers that can be used with Ion Torrent data were identified. Additionally, we showed that the mapping results could be improved by adapting the parameter settings to the error model, for example, by decreasing the indel penalty with SHRiMP2. For Ion Torrent data, our study demonstrated that to be efficient a mapper had to allow indels in the alignments and that the results were more reliable when the mapping algorithm allowed multi-mapped reads. The mutation discovery experiments showed that a sequencing depth of 40*X* was enough to correctly call variants.

## Conclusions

All the different applications that arise from HTS technologies need not have the same mapping characteristics. Some applications may require robust mapping that deals with high error rates while others may require the ability to deal with repeats, for example, when re-sequencing is performed for bacterial variant identification aimed at efficiently detecting mutations and indels. Mappers such as SSAHA2, TMAP, SHRiMP2, or Bowtie2 will support the detection of mutations even at high rates and without the necessity for deep sequencing. In other applications, such as amplicon sequencing to study of repeated motifs (such as CRISPR or IS), the ability to map correctly on repeat regions will be essential and a mapper like SMALT, which performs such tasks very well even though its robustness is not among the highest could be used.

However, for some specific applications, such as the discovery of mutations in viral genomes, mappers such as Bowtie2, segemehl, and SHRiMP2 with strong robustness could be used because accurate mapping of the maximum number of reads, especially the few that bear the mutation, is essential [28].

For some applications, it could be better to use a combination of mappers; for example, in pathogen identification, the strain might be unknown. In this case, SNAP can be used to quickly identify a close reference genome among a set of available genomes, then a more robust mapper can be used to identify mutations or unique reads.

The correct choice of mapper is crucial in HTS data analysis. In this paper, we have presented a benchmark procedure to compare mapping algorithms that are used currently in HTS. Therefore, we introduced a stringent definition of mapping correctness together with a new read simulator, CuReSim, to generate simulated reads with controlled type, rate, and/or distribution of errors along the reads. The read simulator is freely distributed along with a tool to evaluate the mapping quality, CuReSimEval; both are available at http://www.pegase-biosciences.com/tools/curesim/. This procedure was applied to small genomes with Ion Torrent data. Our results do not lead to the selection of a unique, omnipotent mapper but rather show that the choice of mapper has to be application and sequencing technology driven. Our study also demonstrates that a combination of several complementary mappers could significantly improve the mapping step in pipelines. Possible combinations should be tested and evaluated using the same approach. The benchmark procedure presented here greatly helps in the choice of a good mapper for a given application and dataset. This procedure could also be used to evaluate a newly developed mapper or to optimize parameters of an already existing one. An optimized solution for read mapping, adapted to sequencing technology and biological applications, will help compensate for HTS defects.

## Methods

### Mappers

The mappers used in this study were selected from the list given in [2]. The mappers that were explicitly indicated as compatible with Ion Torrent data were selected first; namely, Bowtie2, GSNAP, MOSAIK, Novoalign, segemehl, SMALT, SNAP, and TMAP. Reads generated using the Roche 454 technology have features in common with Ion Torrent reads, so the mapper list was extended to include mappers that were compatible with 454 technology; namely, BWA, BWASW, PASS, SHRiMP2, and SSAHA2. Finally, SRmapper, which is not a sequencing-platform specific mapper, was added. Table 2 lists the 14 selected mappers used in this study and their main features. The main differences between them are the algorithmic approaches and the available options. All the selected mappers index the reference genome, and MOSAIK indexes the reference genome and the reads. The Bowtie2, BWA, BWASW, and TMAP algorithms are all based on the Burrows–Wheeler transform, while the algorithms of the other mappers use hash-tables. TMAP is the mapper that is commonly distributed with the Ion Torrent technology. TMAP uses a series of algorithms (BWA, BWASW, SSAHA2, the super-maximal exact matching algorithm, and the Smith–Waterman algorithm) to map data to an indexed reference sequence. Parallel implementation can greatly decrease execution times for almost all mappers. Most mappers can report all the hits with scores higher than a given threshold; this option is often called the ‘all’ mode report. SRmapper can be run only in the ‘all-best’ mode, which means it reports all hits with the best score. BWASW and SNAP can only be run in ‘any-best’ mode, which means they report only one random hit from among the best hits. The selected mappers were run with their default parameters, except those for which the reporting mode was set to ‘all’ mode. The number of threads was fixed to 24 for all the parallel implemented mappers. The command lines that were used for each of the mappers are available in Section 1 in Additional file 1.

**Table 2:** Description of mappers used in this study

**Features of mappers used in this study**										
**Name**	**Version**	**Algorithm**	**Mis.**	**Indels**	**Gaps**	**Report**	**Align.**	**Parallel**	**Qual.**	**Ref.**
Bowtie2	2.0.4	BWT	Y	Y	Y	A	G,L	Y	Y	[16]
BWA	0.6.2	BWT	Y	Y	Y	A	G	Y	Y	[17]
BWASW	0.6.2	BWT	Y	Y	Y	AnyB	L	Y	Y	[18]
GSNAP	2012-12-20	HT	Y	Y	Y	A	G,L	Y	N	[19]
MOSAIK	2.1.73	HTR	Y	Y	Y	A	G	Y	Y	[20]
Novoalign	2.08.03	HT	Y	Y	Y	A	G	N	Y	
PASS	2.02	HT	Y	Y	Y	A	G	Y	Y	[21]
segemehl	0.1.4-380	ESA	Y	Y	Y	A	G	Y	N	[22]
SHRiMP2	2.2.3	HT	Y	Y	N	A	G	Y	Y	[23]
SMALT	0.7.0.1	HT	Y	Y	N	A	L	Y	Y	
SNAP	0.15	HT	Y	Y	Y	AnyB	G,L	Y	Y	[24]
SRmapper	0.1.1	HT	Y	N	N	AB	G	N	N	[25]
SSAHA2	2.5.5	HT	Y	Y	N	A	L	N	N	[26]
TMAP	3.2.2	BWT	Y	Y	Y	A	G,L	Y	Y	[27]

The column headed ‘version’ indicates the version of the mapper used in this study; the column headed ‘algorithm’ gives the algorithmic method used in the mapper. BWT, the reference genome was indexed with the Burrows–Wheeler transform; HT, the reference genome was indexed with Hash-Table; HTR, the reference genome and reads were indexed with Hash-Table; and ESA, the reference genome was indexed by enhanced suffix arrays. In the columns headed ‘mis.’ (mismatches), ‘indels’, and ‘gaps’, ‘Y’ indicates the algorithm allows mismatches, indels, and long insertions or deletions, and ‘N’ indicates otherwise. The column headed ‘report’ indicates the available report mode: ‘A’ for ‘all’; ‘AnyB’ for ‘any-best’; and ‘AB’ for ‘all-best’ mode. The column headed ‘align.’ ‘G’ (globally) indicates the reads were aligned end-to-end and ‘L’ locally indicates they were not. In the column headed ‘parallel’, ‘Y’ indicates the algorithm is multi-threaded, and ‘N’ indicates it is not. The column headed ‘qual.’ indicates if sequencing quality is taken into account by the mapper (‘Y’ for yes; ‘N’ for no). Bibliographical references are given in the last column.

### Computational resource requirement and time measurement

Memory consumption was measured by parsing the output of the Unix command ‘top’ every second. The time measurement was obtained using the Unix command ‘time’. The real time corresponds to the elapsed wall-clock time. CPU time, obtained by adding the user and system times, is the amount of time the CPU was actually executing instructions. All the mappers were run on a PC with a 6-core processor (2.40 GHz) with 24 GB of RAM.

### Datasets

Real and simulated datasets were used in this study. Three real datasets obtained from the Ion Torrent Community website (http://ioncommunity.lifetechnologies.com/) were used. The main features of the real datasets, RD_100, RD_200, and RD_400, are shown in Table 1. For some experiments, smaller datasets were required; therefore, 50,000 reads were extracted randomly from the real data to generate the smaller datasets. Four files were generated from each dataset and the mean value was computed.

Simulated data were generated from the complete genome of *Escherichia coli str. K-12 substr. DH10B* [GenBank:NC_010473] using CuReSim. The command lines used for read generation are available in Section 1 in Additional file 1. Three datasets were generated with read lengths specific to Ion Torrent technology: mean lengths of 100 bases, 200 bases, and 400 bases with a standard deviation in length of 10% for each dataset. Ion Torrent technology produces reads with about 1% deletions, 0.5% insertions, and 0.5% substitutions [12,29,30]. Each simulated dataset contained 9 files of 50,000 reads with varying indel and substitution rates: the indel rate varied from 0 to 3% and the substitution rate varied from 0 to 1%. Table 3 shows the 9 files that formed the simulated dataset. Each dataset contained, among the 50,000 reads, 2,500 randomly generated reads.

**Table 3:** Simulated datasets used in this study

**Simulated data**			
**Insertion rate**	**Deletion rate**	**Substitution rate**	**Total error rate**
0	0	0	0
0	0	0.5	0.5
0	0	1	1
0.5	1	0	1.5
0.5	1	0.5	2
0.5	1	1	2.5
1	2	0	3
1	2	0.5	3.5
1	2	1	4

The datasets each contained 50,000 reads with varying indel, substitution and error rates. The indel rate varied from 0 to 3%, the substitution rate varied from 0 to 1%, and the total error rate varied from 0 to 4%.

All the datasets and genomes used in this study can be obtained from the authors upon request.

### Mapper robustness

**Figure 8:**
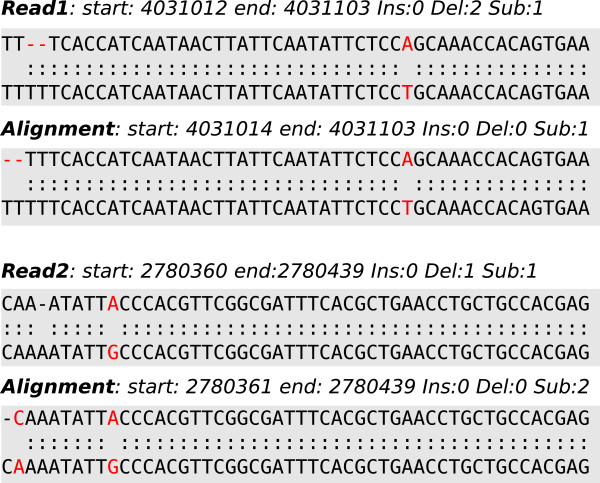
**Reads identified as correctly and incorrectly mapped.** Two representative alignments of simulated reads (read 1 and read 2). produced by a mapper in the special case of indels in homopolymers at the end of an alignment. In each case, the first alignment is the expected alignment for the simulated read with the correct number of insertions, deletions, and substitutions; the second alignment is the alignment returned by a mapper.

To compare the mappers’ robustness, several metrics were computed for the simulated datasets. A read was considered as correctly mapped if among the reported hits at least one hit fitted the following criteria: i) the original start position (*i.e.* the position from which the read is generated) was retrieved, ii) the end position was retrieved, and iii) the alignment produced by the mapper showed exactly the same number of insertions, deletions, and substitutions. Indels in homopolymers at the end of the alignment led to failure to find some correct alignments (the observed start and end positions are not the expected ones). To deal with this special case, a shift for the start and end positions was allowed. The shift gives the number of possible insertions and deletions not considered at the alignment ends. In this case an alignment was considered as correct when the number of insertions (or deletions) in the mapper alignment added to the possible missing insertions (or deletions) was equal to the original number of insertions (or deletions), and the number of substitutions was the same. Figure 8 shows examples of alignments produced by a mapper in the case of indels in this special case. In the read 1 example, the expected alignment starts in 4031012 and ends in 4031103, with two deletions, no insertions; and one substitution; however, the alignment returned by the mapper starts in 4031014, ends in 4031103, and showed only one substitution. Not considering the special case of indels at the alignment ends would have classified this read as incorrectly mapped. However, with our rules, the shift in start positions allowed one deletion at the start of the alignment, meaning that the read was classified as correctly mapped, which reflects reality. In the read 2 example, a shift permitted the addition of one deletion at the beginning of the alignment. However, the number of substitutions was different between the expected and observed alignments; therefore, the read was classified as incorrectly mapped. A read was considered as incorrectly mapped if no hits fitted the three criteria listed above. A read was considered as unmapped if the read was not identified on the reference genome. Precision and recall values were computed as: $\text{precision}=\frac{\text{TP}}{\text{TP}+\text{FP}}$ and $\text{recall}=\frac{\text{TP}}{\text{TP}+\text{FN}}$ with *TP: true positives* being correctly mapped reads, *FP: false positives* being incorrectly mapped reads, and *FN: false negatives* being unmapped reads. The F-measure combines the precision and recall values and was computed as: $F-\text{measure}=2*\frac{\text{precision}*\text{recall}}{\text{precision}+\text{recall}}$ The script to compute these metrics with simulated datasets produced by CuReSim is freely available.

To evaluate the mapper performances on real datasets, the reduced datasets containing 50,000 reads were mapped with each mapper using RABEMA [6] to obtain the percentage of NFI depending on the error rates. RABEMA was run for all the mappers in ‘all-mode’, except for BWASW, SNAP, and SRmapper for which the ‘all-mode’ is not available.

### Study of repeats

A 250,000 bp long artificial genome was generated with five repeats of 500 bp and an error rate of 3%. Using CuReSim, we generated from this genome three sets of 50,000 reads with 0.5% insertions, 1% deletions, 0.5% substitutions, and a mean size of 100, 200, and 400 bases with a standard deviation in length of 10%. This artificial genome was used to evaluate the ability of a mapper to retrieve all locations for a read located in a repeat. A total of 479, 465, and 482 reads for the 100, 200, and 400-base datasets, respectively, were located in one of the 5 repetitions. The number of locations corresponding to a repeat was counted for each of the repeat-located reads.

### Mutation discovery

To evaluate the ability of each mapper to retrieve mutations (*i.e.* true genetic variations within the sample), real and simulated datasets were used with reference genomes in which mutations were introduced artificially at different rates. An in-house script that can take an entire genome as input and return a mutated genome with a given error rate and a file containing the introduced mutations with their type (substitution or indel) and their genome position was used. For the real datasets, three mutated genomes were generated from the complete genome of *Escherichia coli str. K-12 substr. DH10B* with 0.05, 1, and 5% mutations (comprising 90% substitutions and 10% indels). These genomes were used as reference genomes with the real datasets RD_100 and a subset containing 830,000 reads from RD_200. In the same way, three mutated genomes from *Escherichia coli str. K-12 substr. MG16655* [GenBank:NC_000913] were generated to use as reference genomes with a sub-dataset extracted from the RD_400 dataset (595,000 reads). We used sub-datasets for read lengths of 200 and 400 bases to obtain a similar depth of 40*X* for each real dataset. For the simulated dataset, a sub-sequence of 250,000 bp from the *Escherichia coli str. K-12 substr. DH10B* complete genome(from 2,000,001 to 2,250,000) was extracted. From this sub-sequence, we generated three simulated read datasets with CuReSim, with mean lengths of 100, 200, and 400 bp with 10% standard deviation in length and 0.5% insertions, 1% deletions and 0.5% substitutions, and three mutated small genomes with 0.05, 1, and 5% mutations. To obtain a mean depth of about 40*X* for each length set, these 100, 200, and 400 datasets contained 100,000; 50,000, and 25,000 reads respectively. Mutation detection was performed with FreeBayes [15] version 9.9.2-27 (commit id:5d5b8ac). FreeBayes produces a file in Variant Call Format (VCF) that contains all variations. The VCF file was filtered to keep only the variations with a depth of at least 10 reads and a frequency of at least 80%. The ability of the mappers to detect true genetic variations was evaluated by computing precision and recall as follows: $\text{precision}=\frac{\text{CM}}{\text{CM}+\text{IM}}$ and $\text{recall}=\frac{\text{CM}}{\text{CM}+\text{IM}+\text{NM}}$, where *CM* is correctly identified mutations, *i.e.* same type and same or equivalent position as the introduced mutation, *IM* is incorrectly identified mutation, and *NM* is not-found mutation.

## Competing interests

The authors declare that they have no competing interests.

## Authors’ contributions

SC designed the algorithm and wrote the source code, analyzed the project data, and drafted the manuscript. CA and DH supervised the research, carried out the planning and design, contributed to algorithm design and data analysis, and helped to draft the manuscript. YL contributed to the design of the project and to drafting the manuscript. All authors read and approved the final manuscript.

## Supplementary Material

Additional file 1 **Supplementary material.** This PDF file contains supplementary data for this paper. Section 1 - Command lines used for each of the tested mappers and for read generation. Section 2 - Description of CuReSim, the customizable read simulator, and CuReSimEval, the program to evaluate the mapping quality. Section 3 - additional figures. Section 4 - presents additional experiments. Click here for file
