# Supplementary material for: Comparison of mapping algorithms used in high-throughput sequencing: application to Ion Torrent data
Source: BMC Genomics. 2014 Apr 5;15:264. doi: 10.1186/1471-2164-15-264 (PMC4051166; doi:10.1186/1471-2164-15-264)
Supplement: Additional file 1 — Supplementary material. This PDF file contains supplementary data for this paper. Section 1 - Command lines used for each of the tested mappers and for read generation. Section 2 - Description of CuReSim, the customizable read simulator, and CuReSimEval, the program to evaluate the mapping quality. Section 3 - additional figures. Section 4 - presents additional experiments. [file 1471-2164-15-264-S1.PDF]

Supplementary material

1 Command lines

This section gives the command lines used in experiments for each mapper. We have decided to give priority to default settings, except for report-mode which was tuned to report all possible hits and the number of threads was fixed to 24 when the option was available. The command line used to generate simulated reads with CuReSim is also reported.

1.1 Bowtie2

```
> bowtie2-build genome.fasta genome_index
> bowtie2 -x genome_index -U reads.fastq -S result.sam -k 100 -p 24
```

bowtie2-build command is used to index the reference genome and *bowtie2* to map reads to the reference genome. Option $-k$ reports up to 100 alignments per read and $-p$ gives the number of threads to load.

1.2 BWA

```
> bwa index genome.fasta
> bwa aln -t 24 -n $erreur -f result.sai -R 100 genome.fasta reads.fastq
> bwa samse -f result.sam -n 100 genome.fasta result.sai reads.fastq
```

index command indexes the reference genome. *aln* command maps reads. Option $-t$ gives the number of threads. $-n$ is the maximum number of allowed errors ($n = 8$ for read length of 100 bases, $n = 16$ for 200 bases and $n = 32$ for 400 bases). Option $-R$ is used to stop searching when there are more than 100 equally best hits. This step produces a *.sai* file. The *samse* command generates alignment for single-end reads from *sai* to *sam* format with a maximum of 100 occurrences for a read ($-n$ 100).

1.3 BWASW

```
> bwa index genome.fasta
> bwa bwasw -t 24 -f result.sam genome.fasta reads.fastq
```

Genome indexation step is obtained with the *bwa index* command. The *bwasw* command maps reads to the reference genome. BWASW is not able to report several positions for a read ('any-best' mode only). $-t$ flag is to use 24 threads.

1.4 GSNAP

```
> gmap_build -d genome_index -D destination_directory genome.fasta
> gsnap -d genome_index -D destination_directory -t 24 -A sam -M 100 reads.fastq > result.sam
```

Genome index is built with *gmap_index* command. The *gsnap* command is used to map reads with 24 threads ($-t$). The output is a sam file ($-A$ sam) and $-M$ flag is used to report 100 suboptimal hits beyond best hit.

1.5 MOSAIK

```
> MosaikBuild -fr genome.fasta -oa genome.dat
> MosaikBuild -q reads.fastq -out reads.dat -st sanger
> MosaikAligner -in reads.dat -out result_unique.bam -ia genome.dat -p 24 -annpe 2.1.26.pe.100.0065.ann
 -annse 2.1.26.se.100.005.ann  -om
> samtools cat -o result.bam result_unique.bam result_multiple.bam
> samtools view -h -o result.sam result.bam
```

MosaikBuild is used to index the reference genome and reads. The *MosaikAligner* command maps reads onto the genome. Option $-p$ is used to launch 24 threads, $-annpe$ and $-annse$ are used for the neural network filenames. Option $-om$ reports multi-mapped reads in the *multiple.bam* file. $MosaikAligner$ command returns two bam files : one containing one alignment per read and the other containing all the multi-mapped reads. We used samtools to concatenate bam files and convert the resulting file into sam format.

1.6 Novoalign

```
> novoindex genome_index genome.fasta
> novoalign -d genome_index -f reads.fastq -H -x 6 -n 300 -g 20 -o SAM -rEx 1000 -t 250 > result.sam
```

novoindex command builds the genome index and *novoalign* command maps reads onto the reference genome. Option $-H$ hard clips trailing bases with quality less than 2, $-x$ 6 sets the gap extend penalty to 6, $-n$ 300 option truncates reads to 300 before alignment (300 is the maximal value accepted by Novoalign), option $-rEx$ specifies a limit on 1000 alignments reported per read and $-t$ flag sets the maximum alignment score of 250 to be acceptable for the best alignment. These parameter settings were extracted from a study for Ion Torrent data (for more details, see `http://www.novocraft.com/wiki/tiki-index.php?page=Benchmarking+Ion+Torrent+PGM+aligners`).

1.7 PASS

```
> pass -fastq reads.fastq -d genome.fasta -sam -cpu 24
```

pass command indexes the reference genome and maps reads onto it. $-sam$ flag specifies sam format for output file and the $-cpu$ flag fixes the number of threads to launch.

1.8 segemehl

```
> segemehl.x -x genome.idx -d genome.fasta
> segemehl.x -i genome.idx -d genome.fasta -H 0 -q reads.fastq -t 24 > result.sam
```

The first command line indexes the reference genome and the second maps reads onto it. Option $-H$ 0 is set to report all hits and the $-t$ flag to use 24 threads.

1.9 SHRiMP2

```
> gmapper reads.fastq genome.fasta -o 100 -N 24 -Q --qv-offset 33 > result.sam
```

gmapper command indexes the reference genome and maps reads. Option $-O$ 100 is for reporting a maximum of 100 hits per reads, $-N$ 24 is to launch 24 threads, $-Q$ is to use fastq format in input and –qv-offset 33 to interpret quality in fastq input as PHRED+33.

1.10 SMALT

```
> smalt_x86_64 index genome_index genome.fasta
> smalt_x86_64 map -n 24 -d -1 -o result.sam genome_index reads.fastq
```

index command indexes the reference genome. *map* command maps reads onto reference with option $-n24$ to launch 24 threads. Option $-d$ -1 means that all alignments that have scores above the default threshold are reported.

1.11 SNAP

```
> snap index genome.fasta genome_index
> snap single genome_index reads.fastq -o result.sam -t 24 -M -F a -d $erreur
```

index command indexes genome. *single* command maps single reads onto reference genome with 24 threads ($-t$ flag), $-M$ indicates that CIGAR strings in the generated SAM file should use M (alignment match), $-Fa$ is a filter output (a=aligned only) and $-dn$ is maximum edit distance allowed per read ($n = 8$ for read size of 100 bases, $n = 16$ for 200 bases and $n = 32$ for 400 bases). Note that SNAP can only be run in 'any-best' mode.

1.12 SRmapper

```
> buildindex { genome.fasta } genome_index.sqn
> align genome_inedx.sqn { reads.fastq } result.sam -p 100 -a 100
```

buildindex command indexes the reference genome and *align* command maps reads. Option $-p$ 100 is to print a maximum of 100 alignments per read and option $-a$ 100 is to set the maximum number of alignments with equal numbers of mismatches to consider per quarter. SRmapper can only report all-best hits.

1.13 SSAHA2

```
>ssaha2 -454 -output sam -outfile result.sam genome.fasta reads.fastq
```

After several tests, we decided to run SSAHA2 with option tuned for 454 reads which implies options -skip 3 -seeds 2 -score 30 -sense 1 -cmatch 10 and -ckmer 6.

1.14 TMAP

```
> tmap index -f genome.fasta
> tmap mapall -n 24 -f genome.fasta -r reads.fastq -v -Y -u -a 3 -s result.sam -o 0 stage1 map4
```

mapall command maps reads onto genome indexed with *index* command. This command has one stage ($stage1$) using $map4$ algorithm which is a variation of the super-maximal exact matching algorithm. Option $-Y$ includes flow space specific SAM tags when available, option $-v$ prints verbose progress information, option $-u$ specifies to randomize based on the read name, option $-a$ 3 outputs all alignments (i.e. 'all' mode), option $-n$ 24 launches 24 threads and option $-o$ 0 returns output file in SAM format. This command line is the one used by Ion Torrent analysis work flow, except for option $-a$. By default, $-a = 1$ corresponding to 'any-best' mode.

1.15 Read generation with CuReSim

```
> java -jar CuReSim.jar -f genome.fasta -n 47500 -r 2500 -m #size -sd #sd -d #del -i #ins -s #sub
```

This is the general command line used to generate all simulated datasets of this study with CuReSim. $genome.fasta$ can be DH10B.fasta, MG1655.fasta, repeat_genome.fasta or subDH10B.fasta depending on experiment. Option $-n$ 47500 is to generate $47,500$ reads and $2,500$ random reads (option $-r$). Read length (option $-m$) and standard deviation (option $-sd$) is respectively equal to 100 and 10, 200 and 20, 400 and 40. Deletion rate $\#d$ varies from 0 to 0.02. Insertion rate ($\#ins$) and substitution rate ($\#sub$) vary from 0 to 0.01.

2 CuReSim : a customized read simulator

CuReSim (Customized Read Simulator) is a tool which generates synthetic next-generation sequencing reads, supporting read simulation for major letter-base sequencing platforms. CuReSim is developed in Java and is distributed as an executable jar file. CuReSim can be freely downloaded at
`http://www.pegase-biosciences.com/tools/curesim/`.

2.1 Overview

The main features of CuReSim include :
- genome (fasta) or read (fastq) as input file
- choice between several error distributions
- particular attention has been paid to special cases for which several introduced errors in the same read can result in a lower number of errors than expected due to compensatory changes
- generation of diagrams to know exactly the simulated error model (R is required)

2.2 Methods

CuReSim runs in four steps.

Step1 : Input file pre-processing

When the input file is a genome in FASTA format, the first step uniformly draws random genome positions to generate reads without errors from both strands. The read size is normally distributed with a mean and variance which is user-defined. When the input file already contains reads in FASTQ format, the pre-processing step consist in checking format and computing some values such as number of bases and the mean length. At the end of this step, a set of reads without errors is available.

Step2 : Indel generation

In the second step, CuReSim introduces insertions and deletions in reads without errors generated in step1. The insertion and deletion rates are independent and user-defined. Indels can be uniformly drawn along the reads ($option - ui$). They can also be preferentially introduced in homopolymers. In this case, an iterative algorithm mostly introduces indels in the longer homopolymers.

Step3 : Substitution generation

Substitution rate is user-defined. Substitutions can be uniformly drawn ($option - us$) or follow an exponential distribution depending on read position, *i.e.* substitution probability increases toward the end of the read.

Step4 : Correction step

We paid particular attention to special cases for which several introduced errors in the same read can result in a lower numbers of errors than expected due to compensatory changes. For example, deletion of an inserted base is forbidden, as is the substitution of an insertion. However, in some cases, introducing a succession of n mutations can finally lead to a minimal edit distance different from the number of introduced mutations n. The edit distance between two strings is defined as the minimum number of edit operations needed to obtain one string from the other one, with the basic edit operations being insertion, deletion, or substitution of a single character. Below are described two simple examples of this case :

```
Example 1:
ATGC -> 1 deletion (T) -> AGC -> 1 substitution (G->T) -> ATC => number of operations = 2
minimal edit distance = 1 (deletion of G between ATGC and ATC)
Example 2:
AAATGAA -> 1 deletion (T) -> AAAGAA -> 1 insertion (G) -> AAAGGAA => number of operations = 2
minimal edit distance = 1 (substitution T->G between AAATGAA and AAAGGAA)
```

In theory, the number of introduced mutations during simulation should be equal to the minimal edit distance between the read without error and the final read with mutations. We added a step to correct the number of introduced errors with those corresponding to the minimal edit distance. In the case of read simulation, it is important to know exactly the error model of simulation in order to estimate and compute correct values in further analysis. This step can be time consuming. It can be skipped with $option - skip$.

2.3 Output files

Three types of output files are generated : output fastq file, log file and diagrams (optional).

Output fastq file

This file contains reads generated by CuReSim with substitutions and indels. The read name encodes several data on simulation process and is of the form :

```
@<#1>_<#2>_<#3>_<#4>_<#5>_<#6>_<#7>_<#8>

1: genome name
2: original position
3: strand (0=forward;1=reverse)
4: random read (0=non-random;1=random)
5: number of insertions
6: number of deletions
7: number of substitution
8: read number (unique within a genome)
```

Log file

log.txt file records program activities. It gives input parameters, real simulation values and the final distribution of reads depending on the number of errors.

Diagrams (optional)

Diagrams are obtained with $option - v$. R software is required (see `http://www.r-project.org/` for more details on R). Four diagrams are created :

– read length distribution
– indel distribution in homopolymers (figure 1)
– substitution rate depending on read position
– distribution of errors in simulated reads

Figure 1 –

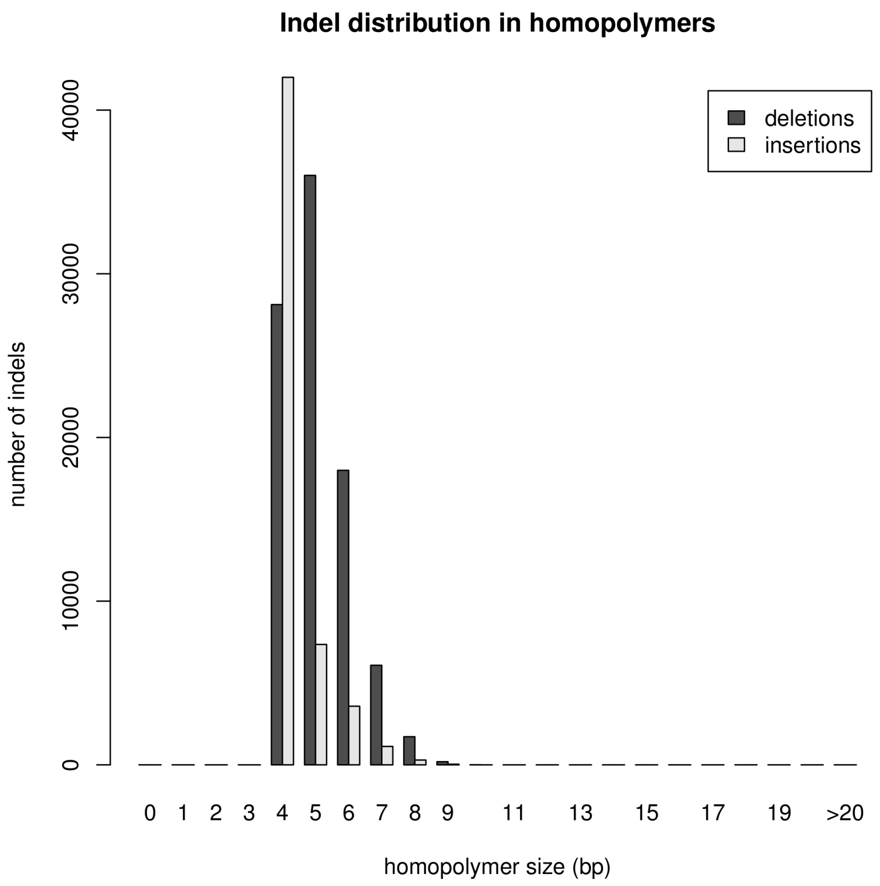

2.4 CuReSimEval : evaluation of read mapping quality

CuReSimEval is developed in Java and is distributed as an executable jar file. This program evaluates the mapping quality for simulated reads simulated by CuReSim.
The main features of CuReSimEval include :

– reads (fastq) and mapping (sam) as input files
– choice between several definitions of mapping correctness
– can be used with a large number of mappers

3 Additional figures

All additional figures cited in the paper can be found in this section.

3.1 Mapper robustness

FIGURE 2 – Precision with varying error rate for simulated reads of 200 bases

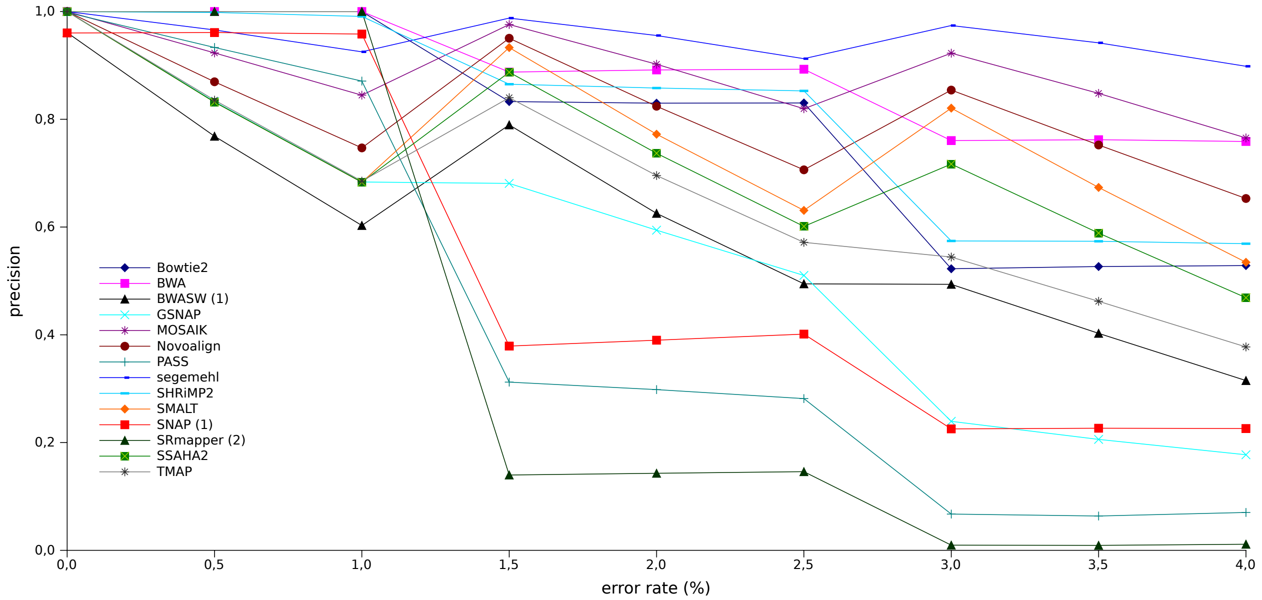

FIGURE 3 – Recall with varying error rate for simulated reads of 200 bases

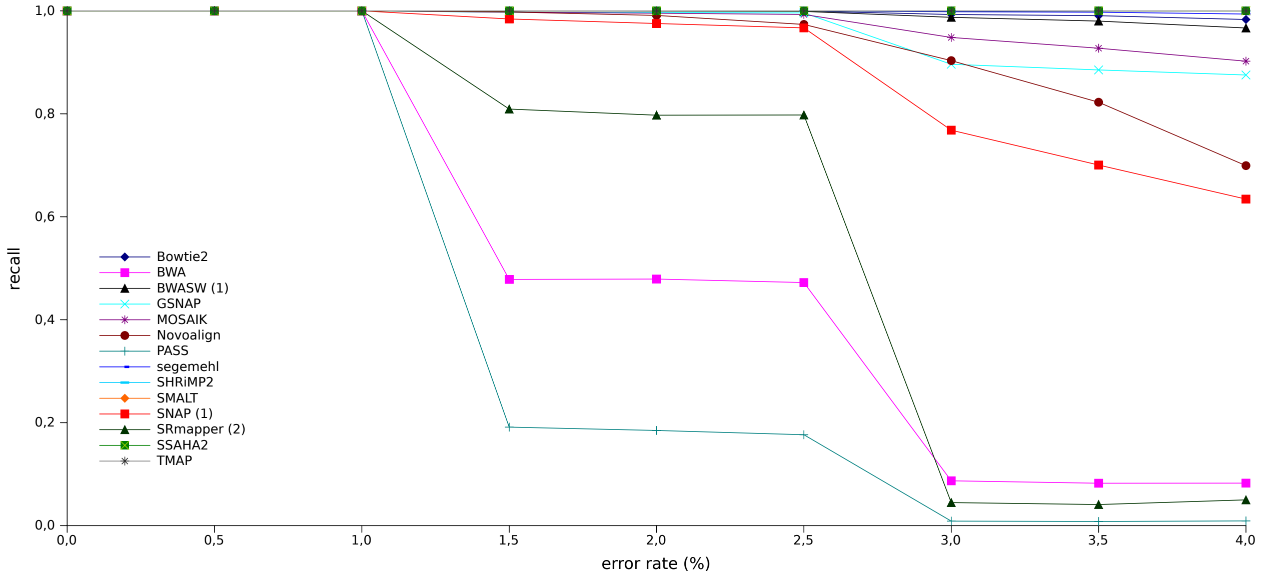

Figure 4 – Percentage of mapped reads with varying error rate for simulated reads of 200 bases

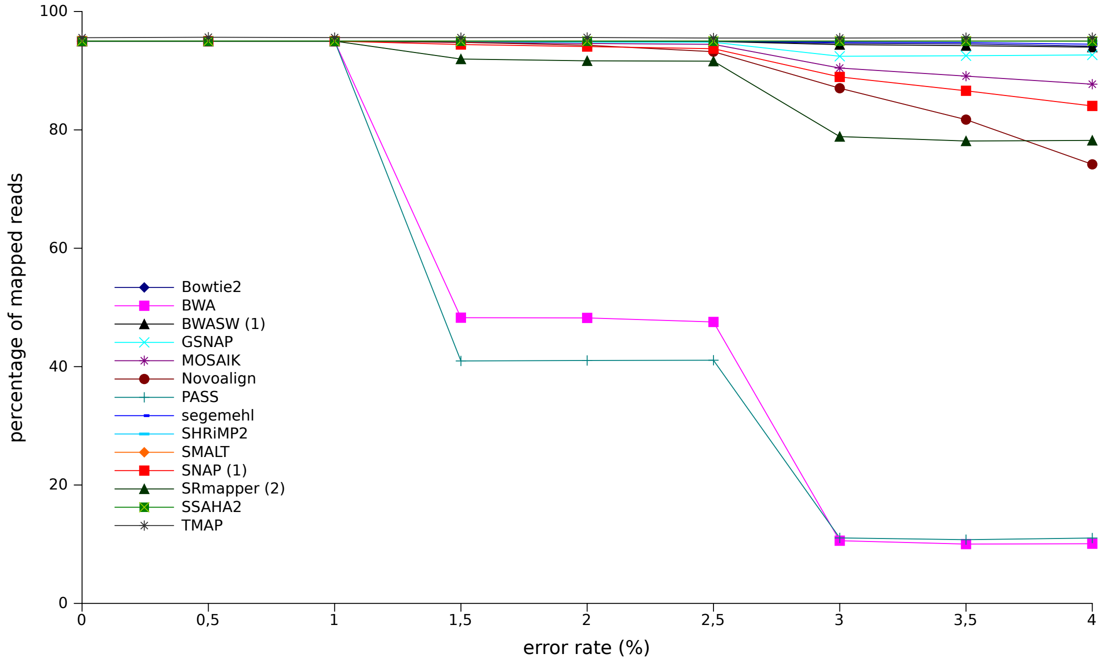

Figure 5 – F-measures with varying error rate for simulated reads of 100 bases

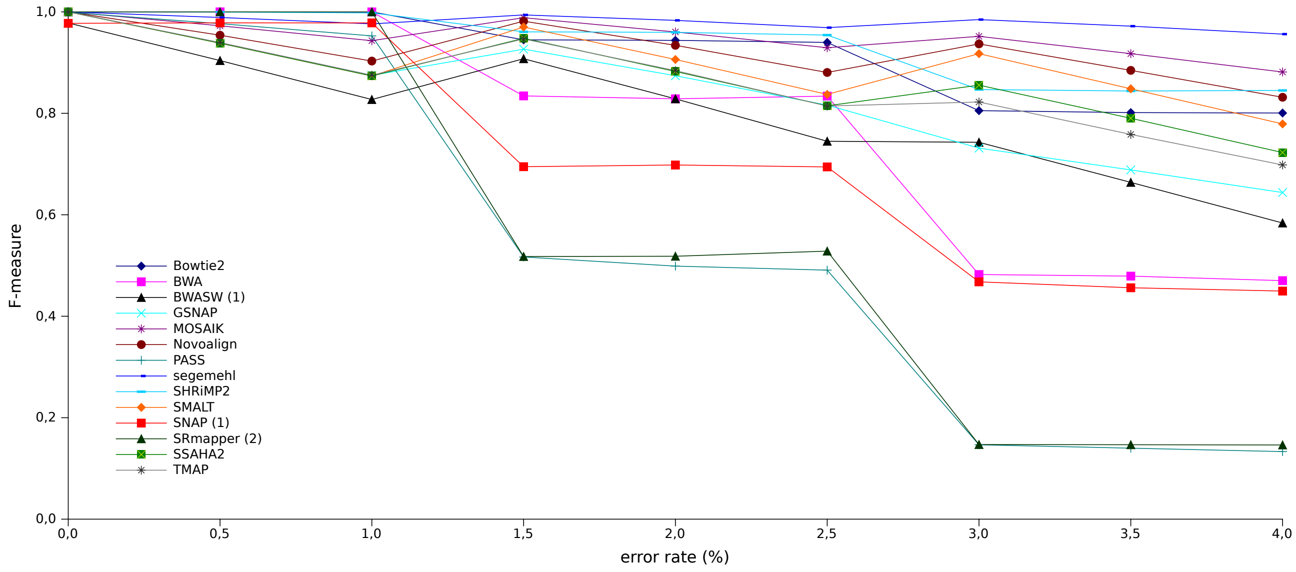

Figure 6 – Precision with varying error rate for simulated reads of 100 bases

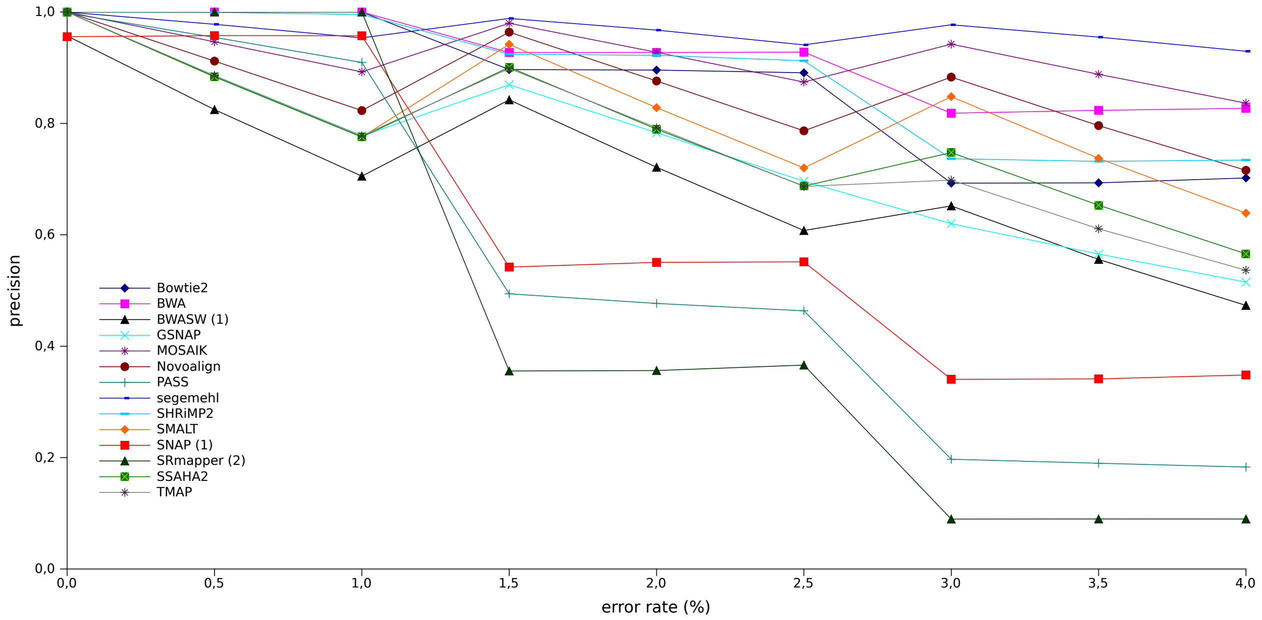

Figure 7 – Recall with varying error rate for simulated reads of 100 bases

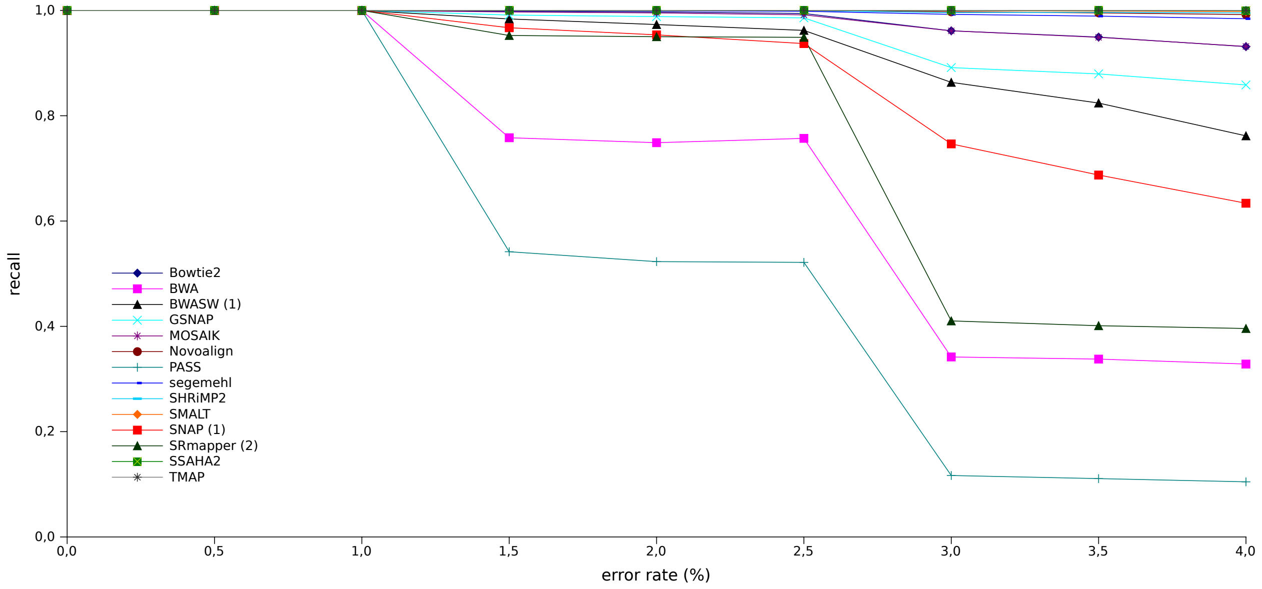

Figure 8 – F-measures with varying error rate for simulated reads of 400 bases

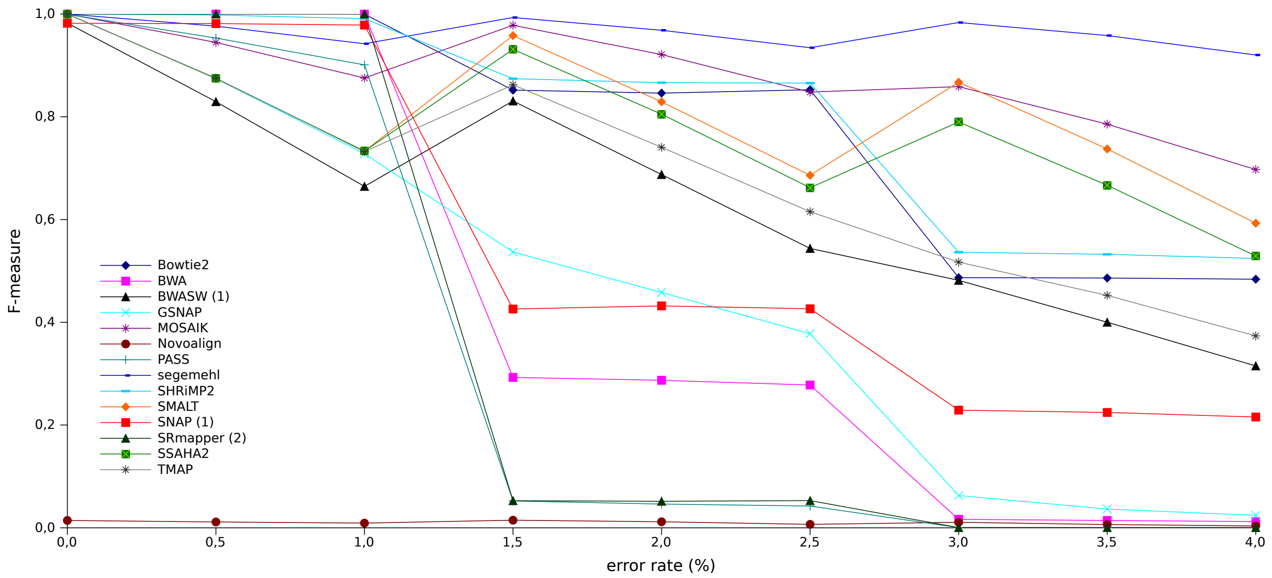

Figure 9 – Precision with varying error rate for simulated reads of 400 bases

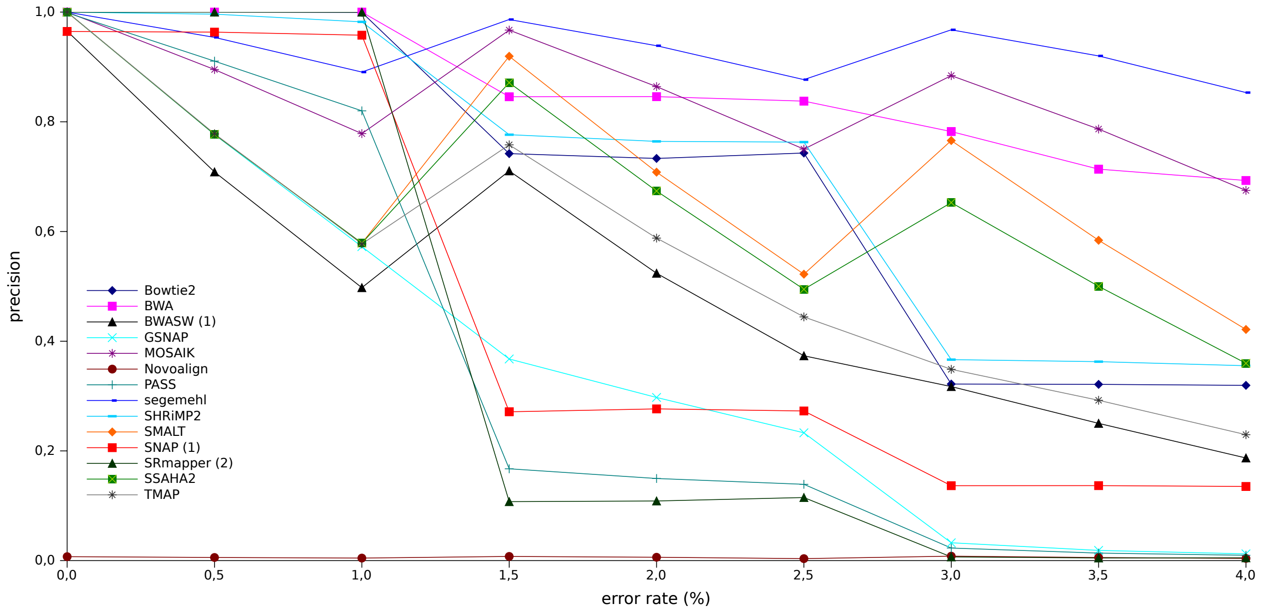

Figure 10 – Recall with varying error rate for simulated reads of 400 bases

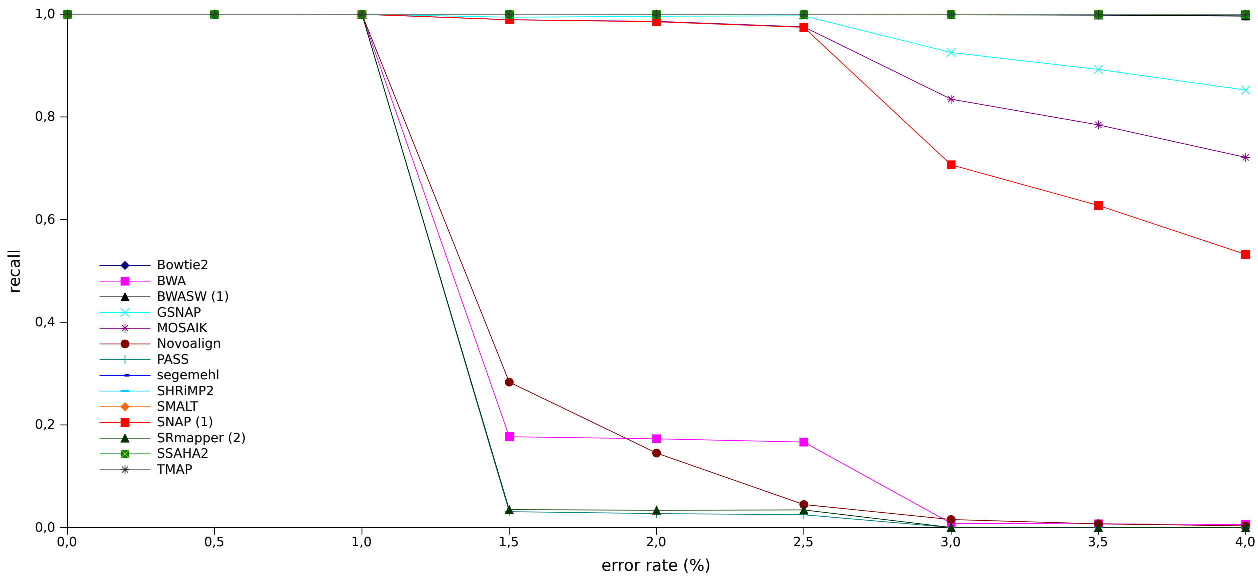

Figure 11 – Normalized found intervals with varying error rate for the RD_100 sub-dataset

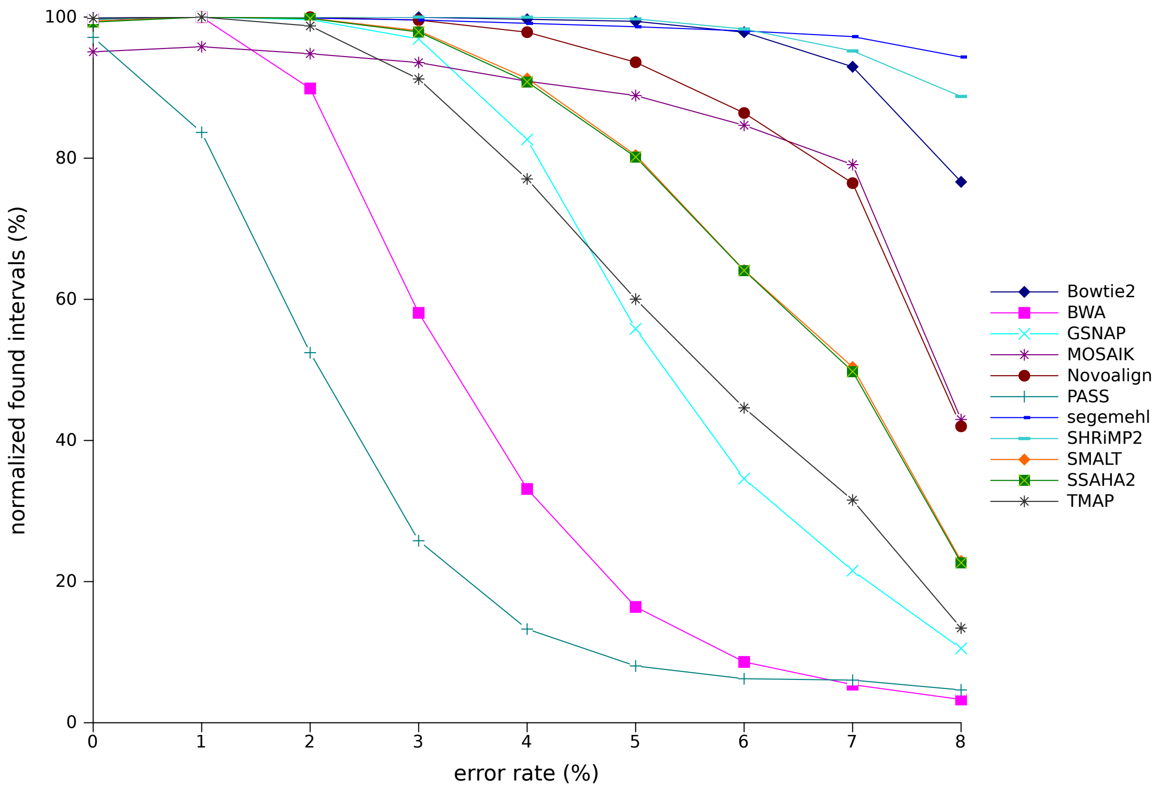

Figure 12 – Normalized found intervals with varying error rate for the RD_400 sub-dataset

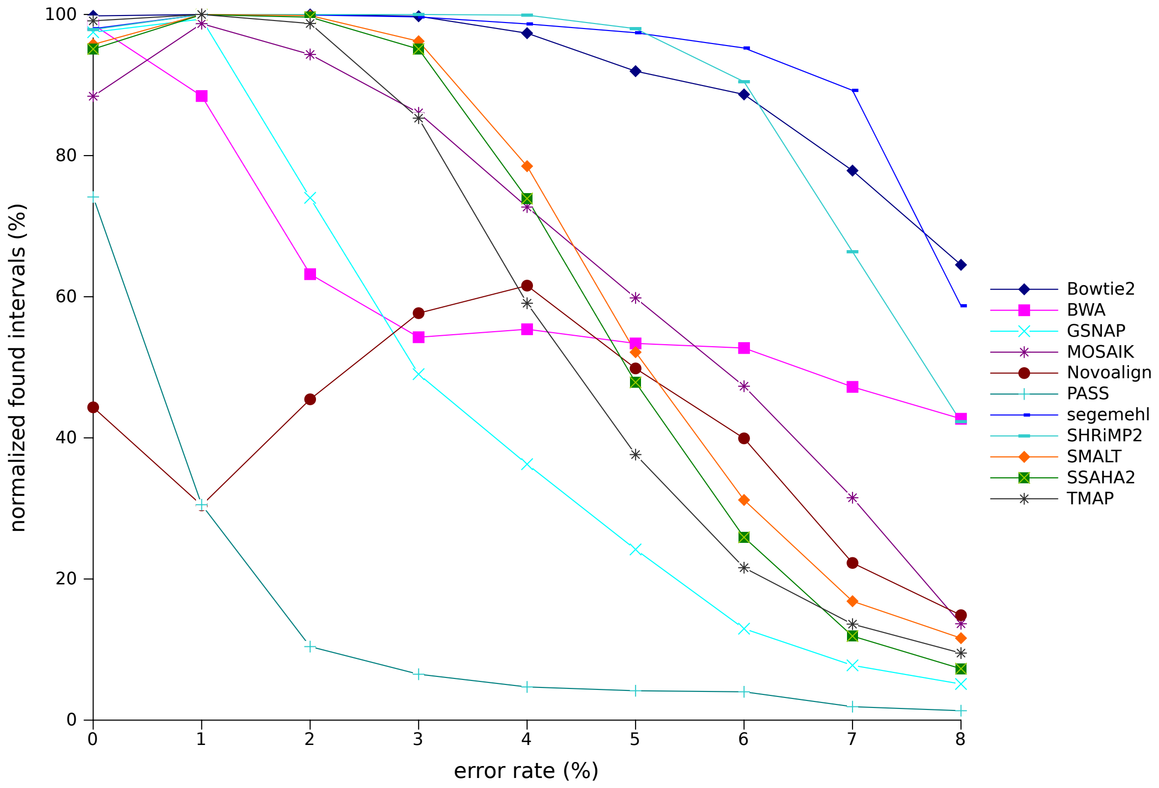

3.2 Study of repeats

Figure 13 – Percentage of repeat-located reads correctly mapped for simulated reads of 100 base length

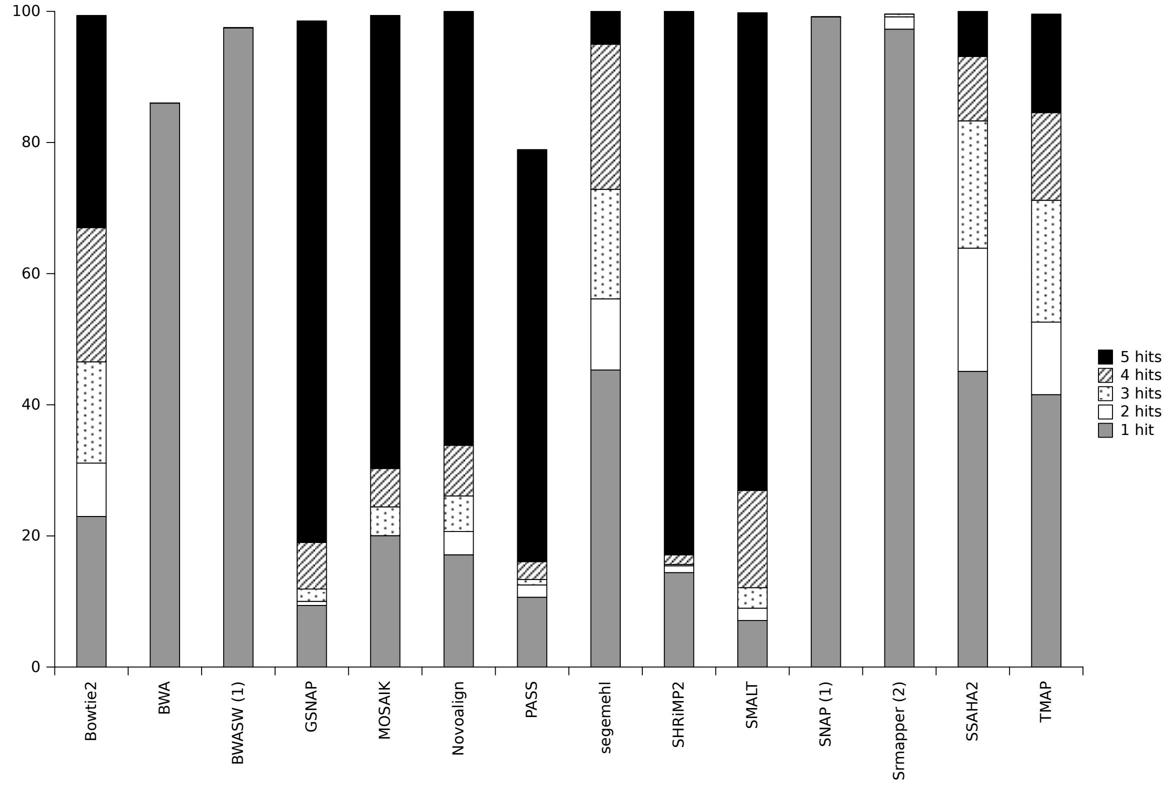

FIGURE 14 – Percentage of repeat-located reads correctly mapped for simulated reads of 400 base length

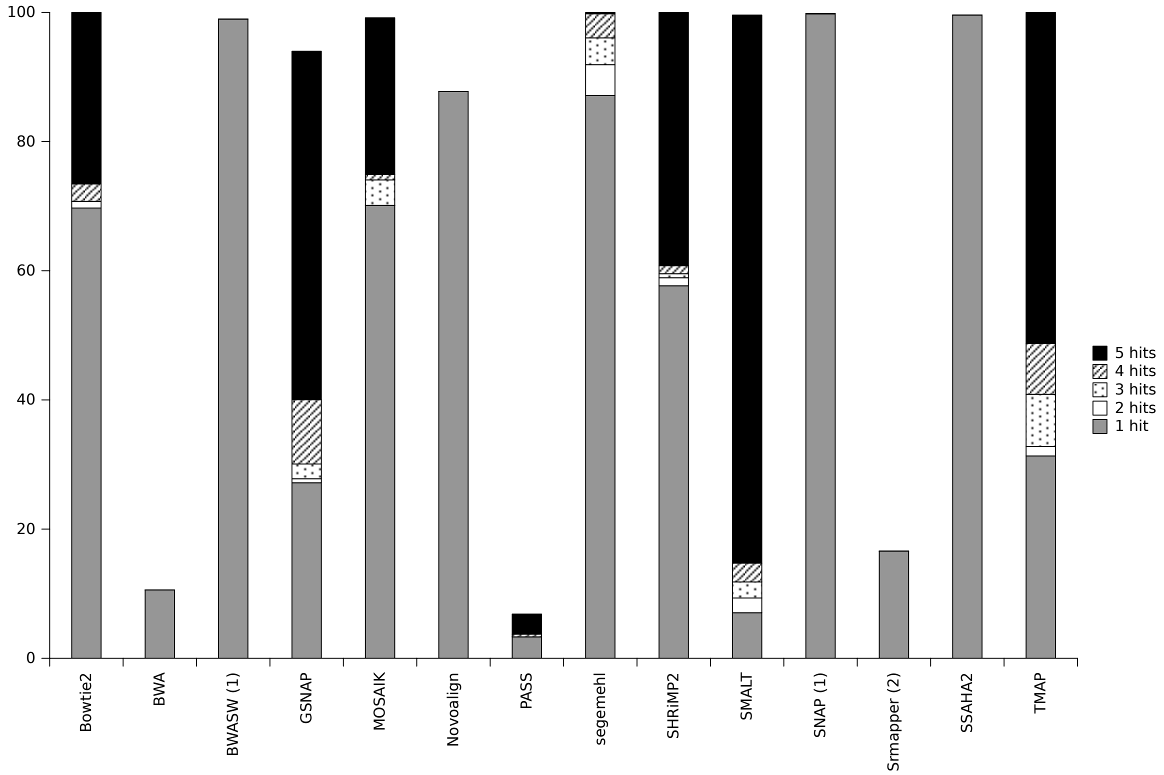

3.3 Mutation discovery

FIGURE 15 – Percentage of mapped reads for RD_200 dataset

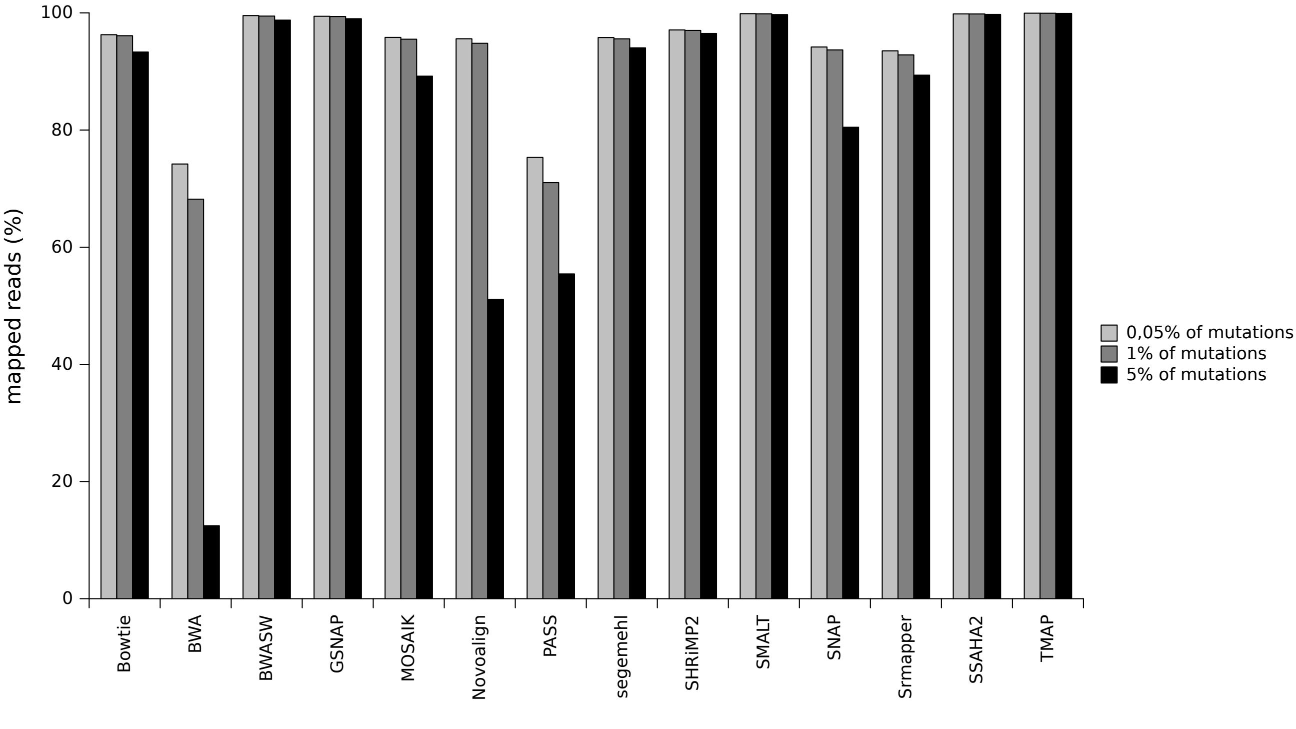

FIGURE 16 – ROC curves for the RD_200 dataset. 0.05 % of mutations are introduced in the reference genome. The mapper showing the lower ROC curve is SRmapper.

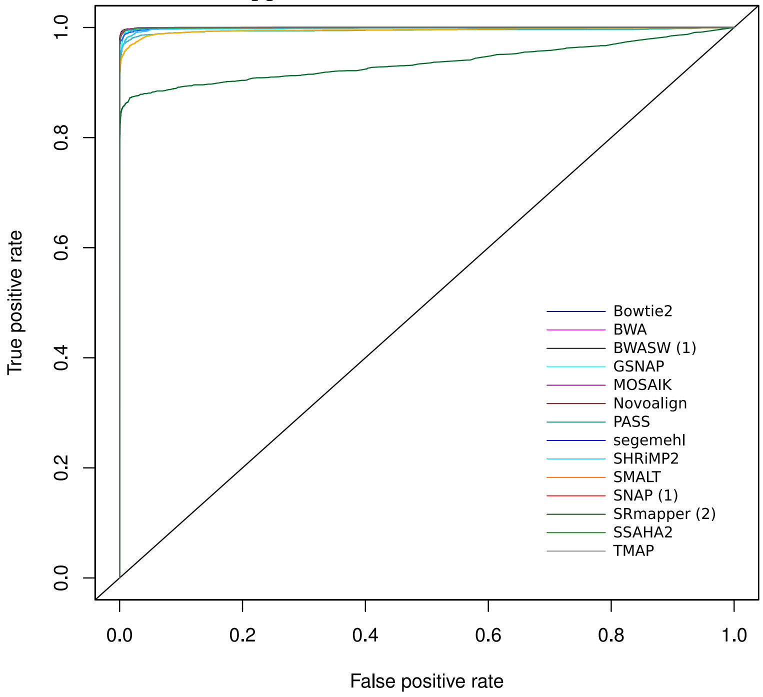

FIGURE 17 – ROC curves for the RD_200 dataset. 1 % of mutations are introduced in the reference genome. The mappers showing the lower ROC curves are respectively SRmapper, GSNAP and PASS.

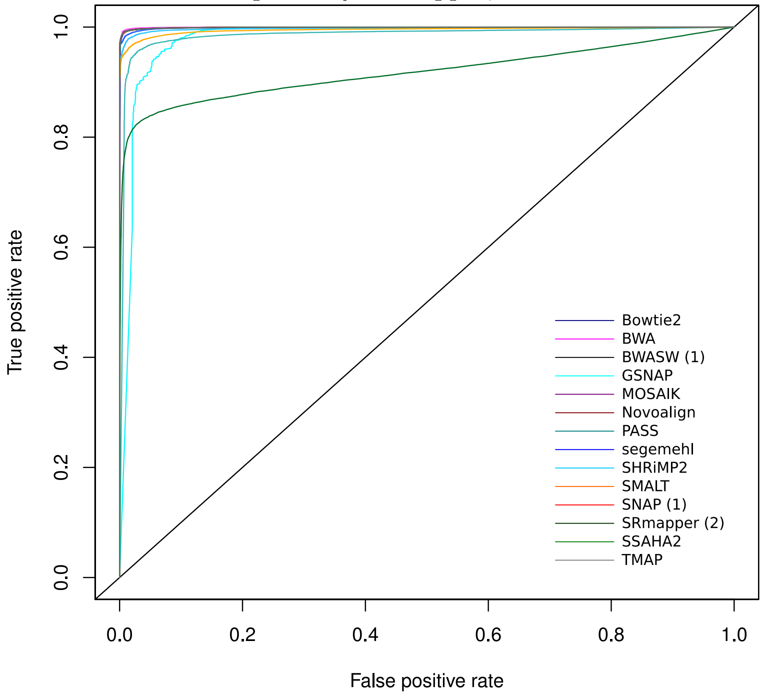

Figure 18 – ROC curves for the RD_200 dataset. 5 % of mutations are introduced in the reference genome. The mappers showing the lower ROC curves are respectively SRmapper, GSNAP and PASS.

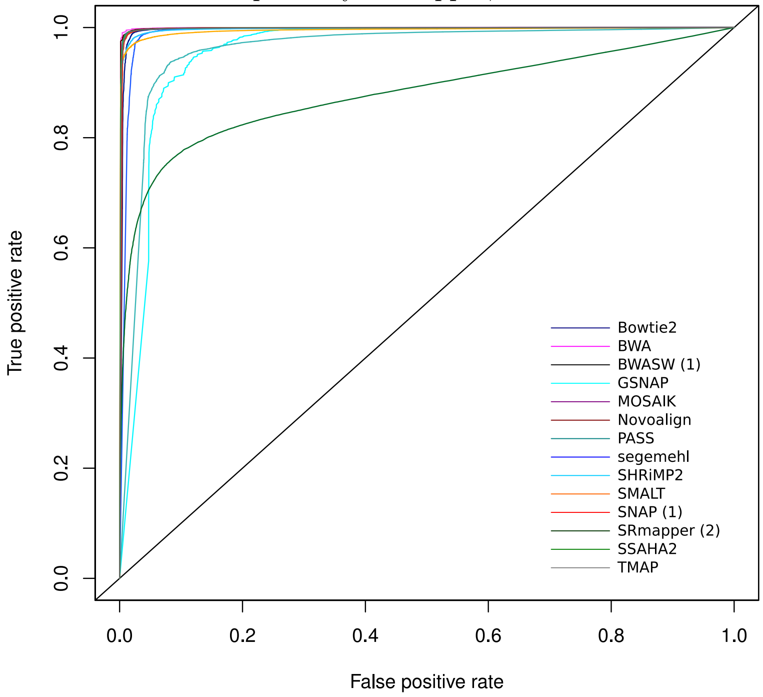

Figure 19 – Accuracy and recall for mutation discovery for the RD_100 dataset

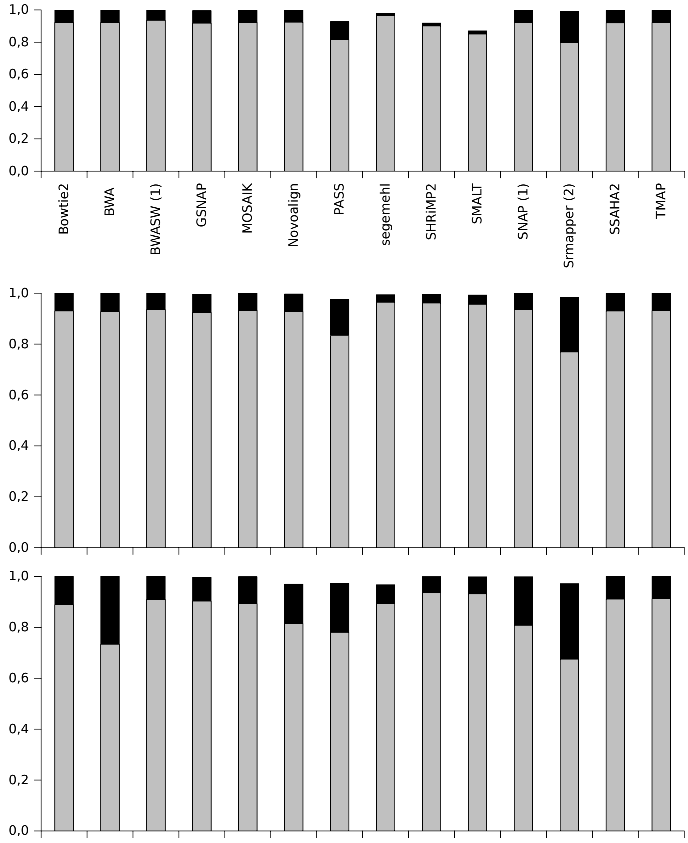

Figure 20 – Accuracy and recall for mutation discovery for the RD_400 sub-dataset

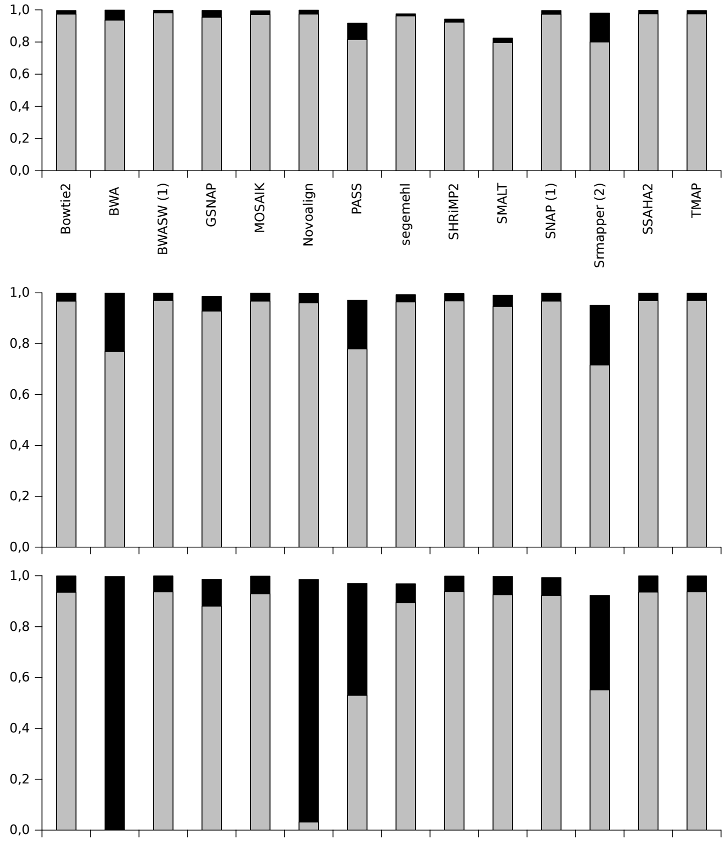

Figure 21 – Accuracy and recall for mutation discovery with simulated reads of 100-base length

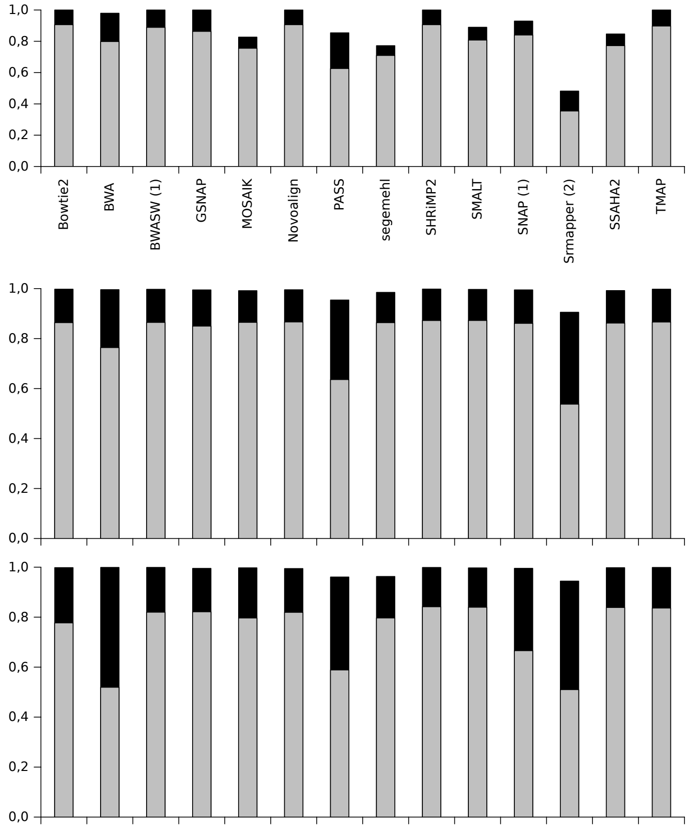

FIGURE 22 – Accuracy and recall for mutation discovery with simulated reads of 200-base length

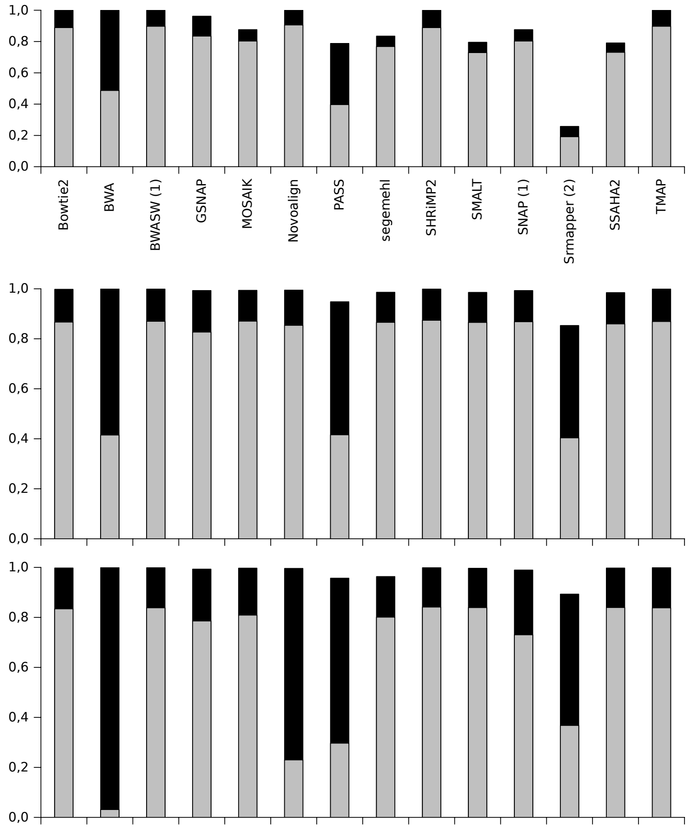

FIGURE 23 – Accuracy and recall for mutation discovery with simulated reads of 400-base length

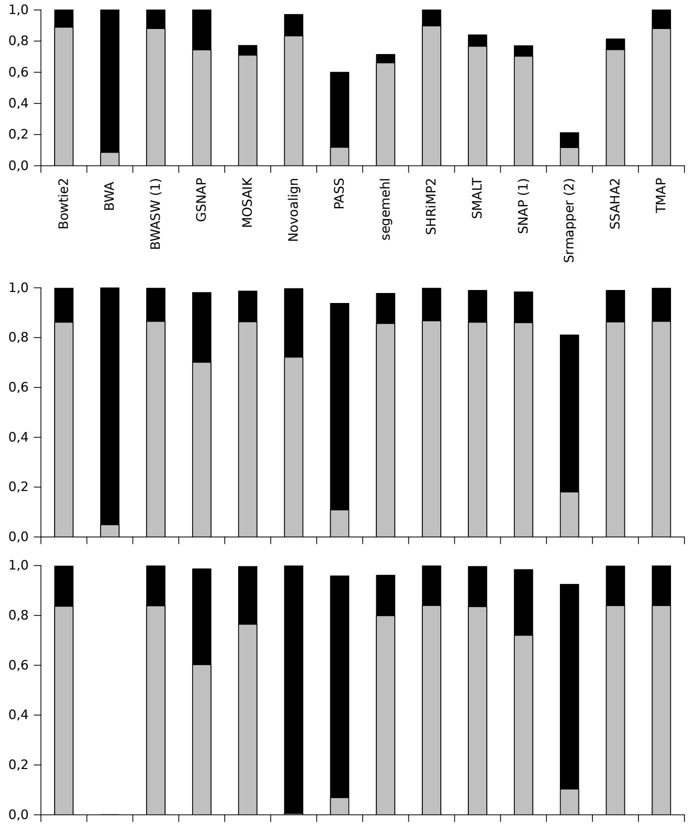

4 Additional experiments

4.1 Mapper robustness : changing the scoring parameters in the alignment step

We changed the gap penalty for two mappers : SHRiMP2 and PASS. For SHRiMP2, the gap open and extension penalties were set to the same penalty as the substitution penalty (*i.e.* -15).

```
> gmapper reads.fastq genome.fasta -o 100 -N 24 -Q -g -15 -q -15 -e -15
 -f -15 --qv-offset 33 > result.sam
```

For PASS, a maximum gap of 8 bases was allowed (option $-g4$) with a gap open and extension penalty of 1.

```
> pass -fastq reads.fastq -d genome.fasta -sam -cpu 24 -g 4
```

Figure 24 shows the results obtained for SHRiMP2 and PASS with the default settings (in blue) and with the modified settings (in red and green).

FIGURE 24 – F-measures with varying mutation rate for SHRiMP2 and PASS with different settings

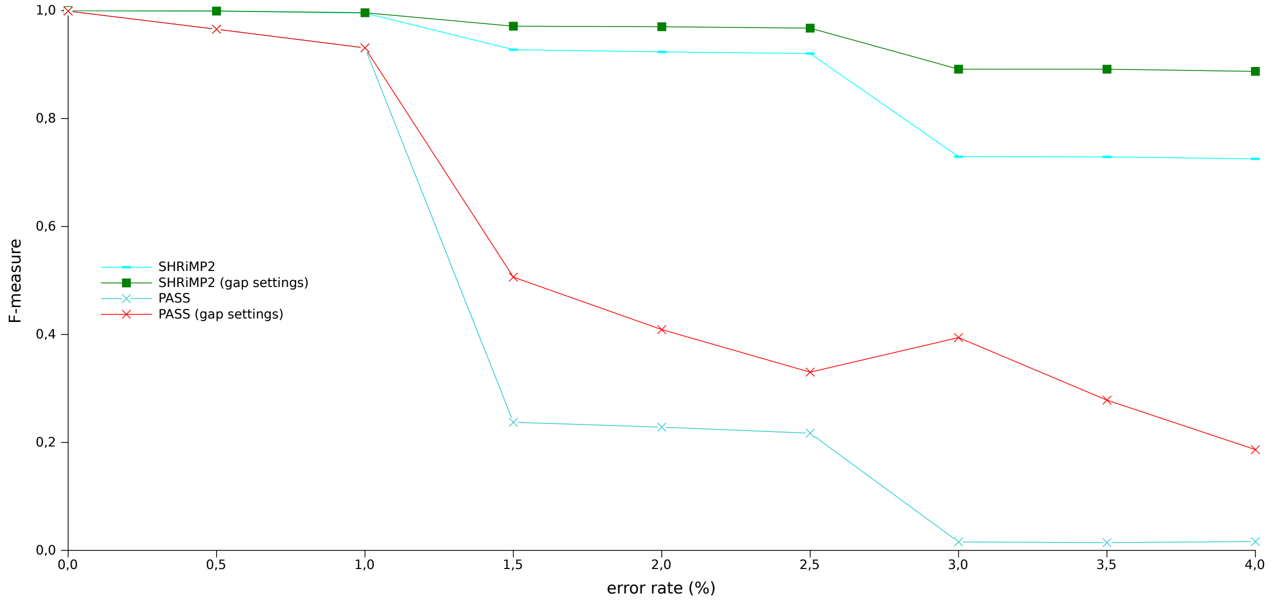

4.2 Mutation discovery : impact of sequencing depth

Thanks to simulated data, we tested the impact of sequencing depth in mutation discovery. The same procedure as that described in the paper was applied with four other read datasets of 200 bases with a mean depth of $20X$, $80X$, $160X$ and $320X$ using SHRiMP2.

TABLE 1 – Precision and Recall for mutation discovery using SHRiMP2 with varying mutation rates in the reference genome and with varying sequencing depth

	$20X$	$40X$	$80X$	$160X$	$320X$
DH10B 0.05%					
precision	0.9123	1.0	1.0	1.0	1.0
recall	0.8189	0.8889	0.8974	0.8974	0.8974
DH10B 1%					
precision	0.9925	0.9990	0.9981	0.9972	0.9972
recall	0.8651	0.8741	0.8730	0.8710	0.8702
DH10B 5%					
precision	0.9945	0.9989	0.9974	0.9983	0.9984
recall	0.8360	0.8409	0.8421	0.8417	0.8409
